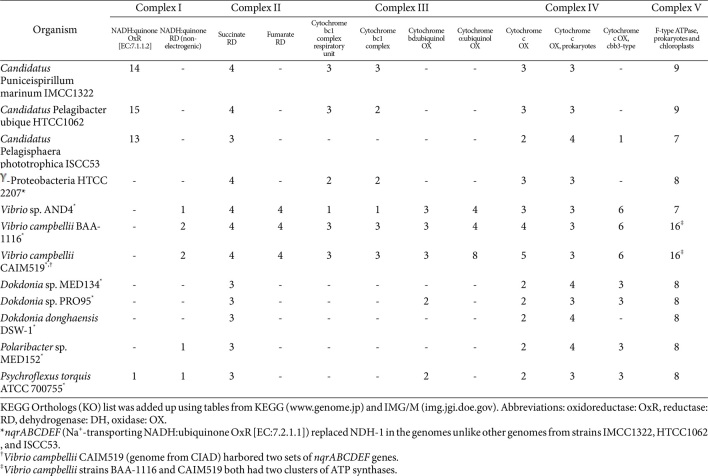# Effect of Light Regime on *Candidatus* Puniceispirillum marinum IMCC1322 in Nutrient-Replete Conditions

**DOI:** 10.4014/jmb.2410.10034

**Published:** 2024-11-27

**Authors:** Hyun-Myung Oh, Ji Hyen Lee, Ahyoung Choi, Sung-Hyun Yang, Gyung-Hoon Shin, Sung Gyun Kang, Jang-Cheon Cho, Hak Jun Kim, Kae-Kyoung Kwon

**Affiliations:** 1 Institute of Liberal Arts Education, Pukyong National University, Busan 48547, Republic of Korea; 2 Department of Pediatrics, Ewha Womans University School of Medicine, Seoul 07804, Republic of Korea; 3 Nakdonggang National Institute of Biological Resources, Sangju 37242, Republic of Korea; 4 Korea Institute of Ocean Science and Technology, Busan 49111, Republic of Korea; 5 Hanyang University ERICA, Ansan 15588, Republic of Korea; 6 Division of Biology and Ocean Sciences, Inha University, Incheon 22212, Republic of Korea; 7 Department of Chemistry, Pukyong National University, Busan 48547, Republic of Korea

**Keywords:** *Candidatus* Puniceispirillum marinum, ATP, inoculum density, light regime, nutrient repletion, proteorhodopsin

## Abstract

Previous studies showed no improvement in bacterial biomass for *Candidatus* Puniceispirillum marinum IMCC1322 under light regimes. Nevertheless, in nutrient-replete cultures with higher inoculating cell densities, strain IMCC1322 exhibited proteorhodopsin photoheterotrophy. Increasing both inoculum size and the amino acid pool can eliminate quorum sensing and starvation responses in strain IMCC1322. Light regimes affected IMCC1322 cultures in stationary/death phases, where cellular ATP levels ranged from 0.0331 to 1.74 mM, with ATP/cell ranging from 13.9 to 367 zeptomoles. In nutrient-depleted conditions, strain IMCC1322 may suffer from excessive protons generated by proteorhodopsin under light conditions. IMCC1322 may tolerate excessive periplasmic protons through ATP-dependent proton pumping and protonation of augmented amino acids. Meanwhile, acid stress could also be mitigated by refining membrane permeability through unsaturation and cyclopropanation of phospholipids. Oceanic bacteria such as IMCC1322 and SAR11 preferred anaplerotic TCA cycles over glycolysis and rely on the Entner-Doudoroff (ED) pathway for growth. Although ATP generation is less efficient in the ED pathway, it offers advantages during rapid growth owing to its strong thermodynamic driving force. The metabolism of IMCC1322 favors gluconeogenesis over glycolysis, aligning with the metabolism of SAR11 reported in previous studies. However, the additional light-driven, PR-dependent ATP synthesis in IMCC1322 is expected to be insufficient to support protein turnover after the log phase, as well as in nutrient-limited conditions. Stable isotope measurements showed no significant differences in the inorganic carbon assimilation between constant light and constant dark cultures in late log phase.

## Introduction

Proteorhodopsin phototrophy in the ocean has been proposed as a globally critical microbial process since proteorhodopsin (PR) was discovered on a SAR86 metagenome [1]. Then, the most abundant marine bacterioplankton, SAR11, revealed the highly functional PR in the original cultivation study [2, 3]. When grown on LNHM (low-nutrient heterotrophic medium) without added organocarbons, PR-bearing SAR11 showed no differential responses between diel and constant dark conditions [2]. Efforts to elucidate proteorhodopsin phototrophy (PRp) in SAR11 have continued but there have been no discernable cultural responses under light regime changes. However, light-mediated ATP production for endogenous carbon respiration was confirmed [4](Table 1). SAR116 and SAR92 bacteria have been successfully cultivated [5, 6], whereas PRp has not been confirmed in these abundant marine bacterioplanktons [7, 8].

PRp under carbon-starved conditions has been reported for a variety of marine microbial taxa in the gram-negative bacteria, including the phyla *Pseudomonadota*, *Bacteroidota*, and most recently, *Verrucomicrobiota*. Phylum *Pseudomonadota* bacteria include *Candidatus* Pelagibacter ubique (SAR11) [2, 4], Gammaproteobacteria HTCC2207 (SAR92) [8], *Vibrio* sp. AND4 [9], *Vibrio campbellii* BAA-1116 [10], *Candidatus* Puniceispirillum marinum (SAR116) [7], and *Vibrio campbellii* CAIM519 [11] (Table 1). The second most-studied phylum *Bacteroidota* includes five cultivated strains from the family *Flavobacteriaceae*; *Dokdonia* sp. MED134 [12, 13], *Dokdonia* sp. PRO95 [14], *Dokdonia donghaensis* DSW-1 [15], *Polaribacter* sp. MED152 [16], and *Psychroflexus torquis* ATCC 700755 [17] (Table 1). Finally, the most recent member of the PR group might be from phylum *Verrucomicrobiota*, *Candidatus* Pelagisphaera phototrophica ISCCC53 [18] (Table 1).

*Dokdonia* sp. MED134 was the first PR-dependent photoheterotrophic bacterium to be identified [12], and its light-induced transcriptional responses have been studied [19]. However, subsequent studies on PRp on other *Flavobactericeae* strains have not been promising, except for that of *Dokdonia donghaensis* DSW-1 [15]. Non-functional PR-bearing Flavobacteria include *Dokdonia* sp. PRO95, *Polaribacter* sp. MED152, and *Psychroflexus torquis* ATCC 700755 [16, 17, 20] (Table 1).

*Vibrio* sp. AND4 (= *Vibrio campbellii* AND4) cultures have been shown to promote survival during starvation and to enhance growth under constant light conditions [9]. However, no growth enhancement was observed under different light regimes in other *Vibrio* culture studies, including those on *V. campbellii* BAA-1116 and *V. campbellii* CAIM519 [10, 11]. *Candidatus* Pelagisphaera phototrophica ISCC53 from phylum *Verrucomicrobiota* showed enhanced growth not in carbon-replete but in carbon-depleted cultures under a diel-cycled light regime where more ATP production might reflect increased sugar uptake [18]. Although the strains were PRp-impaired, previous studies have conducted ATP measurements for *Candidatus* Pelagibacter ubique, *V. campbellii* BAA-1116, and *V. campbellii* CAIM519 (Table 1).

To our knowledge, previous studies on PR-bearing marine bacteria have not supported the highly anticipated molecular function that microbial ecologists hoped for, as summarized in Table 1. Ubiquitous and dominant strains like SAR11 and SAR116, including IMCC1322, have never shown light-enhanced growth under laboratory conditions. However, PRp activity has occasionally been observed in less abundant PR-bearing cultures from *Flavobacteria* and *Vibrio* species (Table 1).

Here we report enhanced PRp under nutrient-replete cultures of strain IMCC1322, which was not expected of previous cultivation studies using nutrient-depleted or carbon-restricted conditions.

Based on previous IMCC1322 culture results with amino acid augmentation [7] and nutrient-replete cultures in this study showing light-enhanced growth, we propose that PRp in IMCC1322 may depend on nutrient-replete conditions. This could support a mechanism of excessive metabolic energy expenditure to outcompete other species. In this context, we will discuss nutrient repletion, cell density, and ATP production under light conditions, including changes in biomass (cell volumes) and intracellular ATP levels during stationary phases.

## Material and Methods

### Setup for Culturing Strain IMCC1322 Under Various Light Conditions

The basal culture medium was 0.5 g/l each of Proteose Peptone No. 3 (BD Difco, 211693, USA), yeast extract (BD Difco, 212750), and casamino acid (BD Difco, 223050) prepared in an aged seawater and described as mPYC [21].

Single colony-picked bacteria were suspended in 1-ml 1x mPYC and incubated in a shaking incubator until they became turbid. Seeding cultures were inoculated into 100 ml and or a larger volume of culture medium and sub-cultured for every 4 or 5 days. Cell morphology and OD_600_ were assessed daily. We used 2% or 1/50 volume of actively growing IMCC1322 culture as the inoculum, unless otherwise stated, and the growing temperature was set at 25°C.

Strain IMCC1322 was inoculated into 2.2-L magnetic spinner flasks (Nalgene Cat. No. 2605-0001) (~500 ml culture volumes; magnetic stirrer set at 50 rpm). Cultures were maintained under constant dark (DD) or constant light (LL) conditions. LL condition was under pure-green light-emitting diodes (LEDs) (wavelength =525 nm) and the intensity of the green LED was with 260 μJ/cm^2^/s. DL cultures were automatically turned on and off throughout the day under 10-h DD and 14-h LL (10:14 D:L) condition.

### Nutrient-Replete Media Test

In this study, nutrient repletion was done by increasing three carbon sources in proportion to the basal culture medium of mPYC components, which was designated as 1x mPYC, 1.25x mPYC, 1.5x mPYC, 1.75x mPYC, 2x mPYC, 2.25x mPYC, and 2.5x mPYC. DD and LL conditions were checked for cultures with different nutrient concentrations. Moreover, the optical density at 600 nm was measured daily. Nutrient-limited conditions in cultures could be achieved using diluted components, *e.g.*, 0.5x mPYC (see below).

### Inoculum Effect Test

DD and LL growth curves were tested across inoculum densities of 2% (1/50 volume), 4% (1/25), and 10% (1/10). Cultures in basal medium (=1x mPYC) and 0.5x mPYC were checked by OD_600_ for growth curves under DD and LL conditions.

### Changing Light Regimes

LL, DD, and DL cultures were assessed using growth curves for IMCC1322 cultures grown in 0.5 x mPYC, 1x mPYC, and 1.5x mPYC.

Light regime change was checked for samples from DD and LL cultures using mPYC medium with 10% (1/10) inoculum. From cultures on the verge of stationary/death phase, sampled cultures from DD and LL conditions changed their light regimes by relocating to LL and DD incubators, respectively.

### Total Bacterial Cell Counts and Size Measurements

Growth curves with direct cell counts were obtained with staining method using DAPI (4’,6-diamidino-2-phenylindole; Sigma Aldrich, #D9542, USA) [22]. DAPI-stained cells were counted using an epifluorescence microscope (Nikon 80i, Nikon, Japan), and ImageJ software [23] was used to measure cells. Lengths, widths, and areas of the stained-bacterial cell images were used for bacterial volume calculations (refer to supplementary text for details).

### ATP Assay Using Luciferin/Luciferase Kit

The ATP contents of the harvested cells were determined using the Enliten Luciferin/Luciferase Kit (Promega, USA), and the relative luminescence unit (RLU) values were measured using a GloMax 96-Well Microplate Luminometer (Promega). Buffer A for cell suspension (50 mM PIPES pH 6.9, 40 mM NaCl, 2 mM KCl, 2 mM MgCl_2_, 1 mM CaCl_2_, added 3mM dithiothreitol just before use) and ATP assay buffer B (20mM Tris/acetate, pH 7.75) were prepared. Samples of cells in 1.0 ml volumes were harvested in a microcentrifuge and washed by resuspending in buffer A. Pellets were resuspended in 300 μl buffer A and disrupted by freeze-thaw 3 times in liquid nitrogen. Thereafter, lysed 100 μl samples were mixed with 900 μl buffer B. Then, 96-Well Sterile Microplates (Promega) loaded with 100 μl of diluted samples were overloaded by injecting 100 μl rLuciferase/Luciferin (rL/L) reagent. A 2-s time delay after rL/L reagent injection and a 10-s RLU signal integration time were used for data collection. The relative luminescence level was then converted into moles of ATP by extrapolating the RLU of each sample with standard curve of ATP (0.0, 1.0x10^-7^,1.0x10^-6^,1.0x10^-5^, 1.0x10^-4^, 1.0x10^-3^ moles). Molarities of ATP derived from RLUs were divided by DAPI-counted-cell numbers or the cellular volumes, to obtain moles of ATP/cell or ATP in molarity (M).

### ^13^C-Bicarbonate Labeling of Cultures and Stable Isotope Ratio Measurement

Cultures under LL or DD conditions were grown in 1x mPYC furnished with or without ^13^C-NaHCO_3_ (CLM-441-5, Cambridge Isotope Laboratories, Inc., USA). Cultures (OD_600_ ~ 0.08) were harvested in 50-ml conical tubes in an Eppendorf 5804R centrifuge (14,000 ×*g*, 30 min at 4°C) and then resuspended with autoclaved seawater (the component of 1x mPYC). Harvested and washed cultures were freeze-dried and maintained at -70°C in a deep freezer until analysis. Stable isotope studies were performed using an elemental analyzer (CHNS_O, Euro Vector, Italy) combined with a stable isotope mass spectrometer (Isoprime 100, Isoprime Ltd., UK).

The delta (δ) notation in parts per thousands (‰) relative to urea was used as the control. The stable isotope ratios were expressed as follows:

δ^13^ - C or δ^15^ - N (‰) = [(^13^C/^12^C or ^15^N/^14^N)_sample_ / (^13^C/^12^C or ^15^N/^14^N)_standard_ - 1] × 1000.

### Bioinformatic Analysis of Amino Acid Profiling in *Candidatus* Puniceispirillum marinum IMCC1322

The amino acid profiling began by obtaining transcriptome data from a previous study [24], assuming that mRNA levels parallel protein polymerization [25]. Coding sequences corresponding to counted mRNA sequences were downloaded in amino acid FASTA format from GenBank. Each amino acid FASTA file was processed using the amino acid composition analysis program, aacomp [26]. The amino acid compositions and gene count numbers from each condition, derived from RNAseq data, were summarized to determine the relative distribution of amino acids. These distributions were visualized using radar charts in Microsoft Excel.

For further investigation, we used the Kyoto Encyclopedia of Genes and Genomes (KEGG) [27] to check the predicted amino acid biosynthesis for *Candidatus* Puniceispirillum marinum (https://www.kegg.jp/pathway/mapno=01230&orgs=apb). The predicted amino acid biosynthetic capabilities were demarcated with previous culture results [7] and amino acid concentrations in 1x mPYC. Each amino acid and inorganic ions were calculated according to the BD Difco Manual of Microbiological Media [28].

## Results and Data

### Effect of Nutrient Repletion on Cultures

**Fig. 1:**
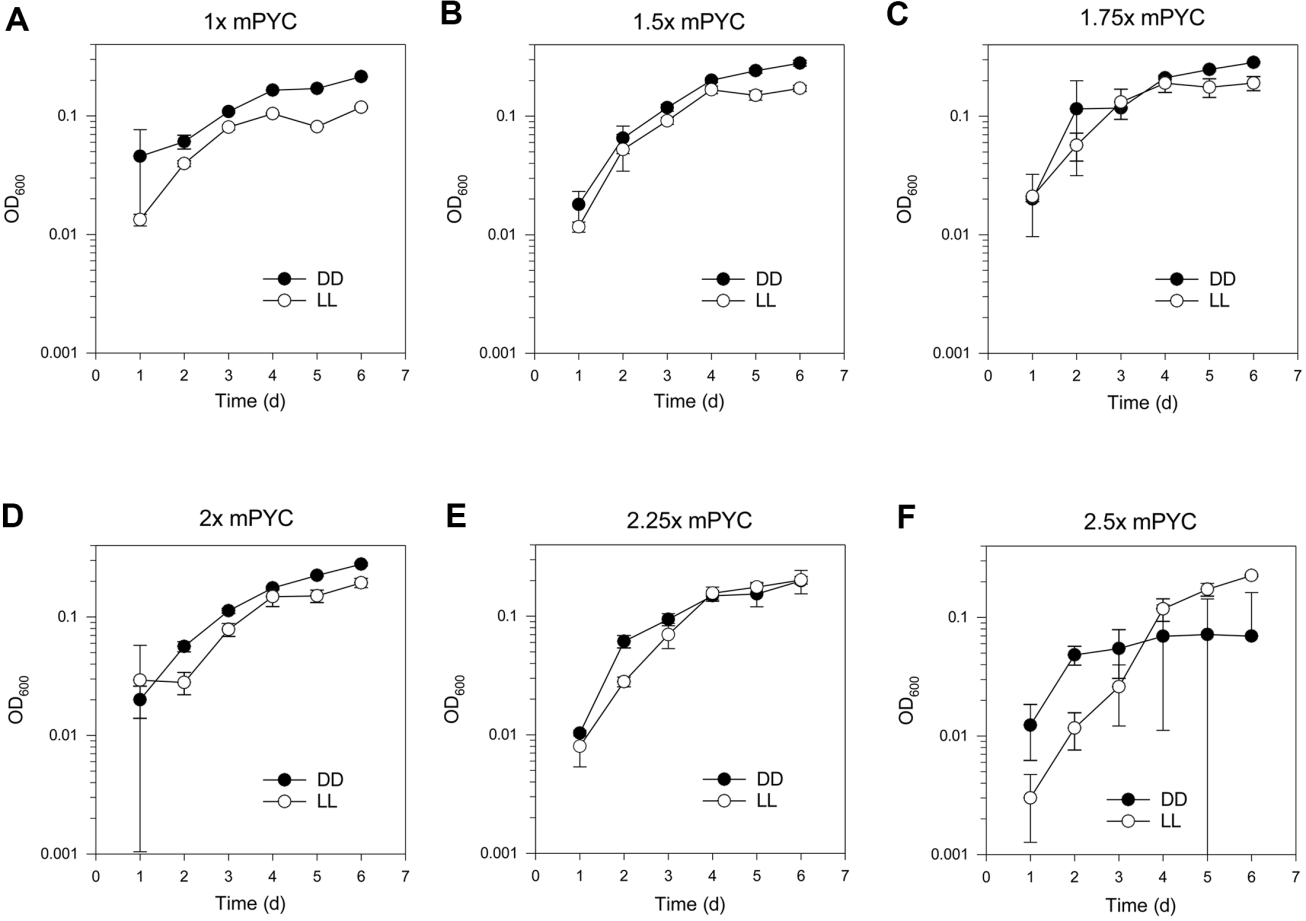
Effect of nutrient-augmented conditions on IMCC1322 cultures. OD_600_ was used for drawing growth curves. From 1x mPYC up to 2.5x mPYC growth curves for DD and LL conditions. Initial inoculum density was 2% or 1/50 volume of the culture. At 4 days post-inoculum higher OD_600_ values under DD conditions than LL conditions (**A-E**), which was not under DD condition for 2.5x mPYC (**F**).

Absorbance (OD_600_) measurements were used to construct growth curves (from 1x mPYC up to 2.5x mPYC), which were obtained for both DD and LL conditions, with the initial inoculum being 2% or 1/50 of the culture volume (Fig. 1). The OD_600_ values under LL condition were higher than the OD_600_ under DD condition in 2.5x mPYC at 6 days post-inoculum (Fig. 1F). The OD_600_ values under LL condition were nearly identical with the OD_600_ under DD condition in 2.25x mPYC at 6 days post-inoculum (Fig. 1E). Other nutrient conditions resulted in higher OD_600_ values under DD conditions at 6 days post-inoculum (Fig. 1A-1D).

### Effect of Inoculum Density on Cultures

**Fig. 2:**
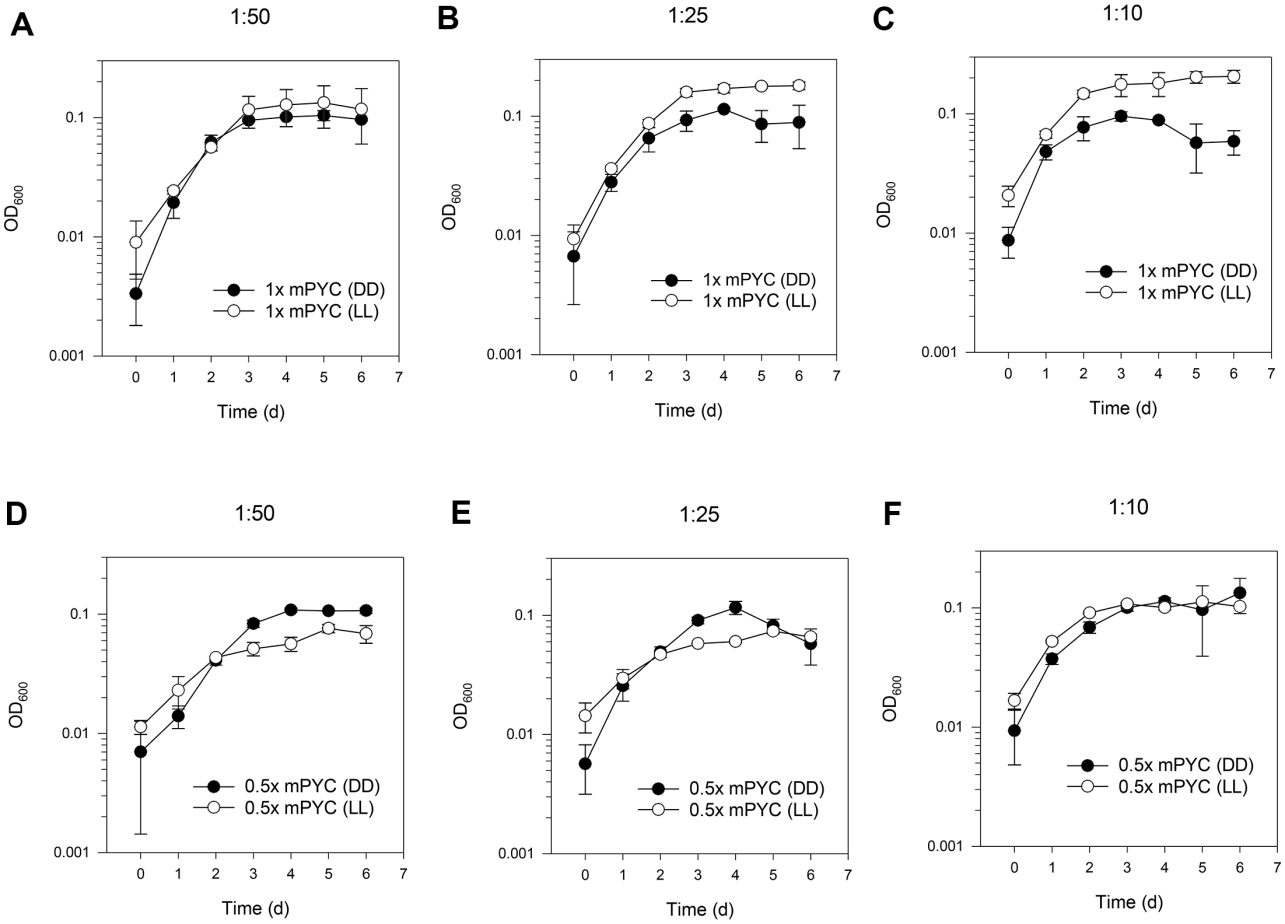
Effect of inoculum density on IMCC1322 cultures. In the growth curves with initial inoculum density of 2 , 5, and 10% of the culture volume, the OD_600_ values of LL cultures outperformed those of DD cultures in 1x mPYC medium in an inoculum density-dependent manner (**A-C**). However, DD cultures in 0.5x mPYC were not surpassed by LL cultures independent of the inoculum density (**D-F**).

The OD_600_ values of LL cultures outcompeted the values of DD cultures in 1x mPYC medium as the inoculum density increased (Fig. 2A-2C). DD cultures in 0.5x mPYC were not overtaken by LL cultures in the growth curves (Fig. 2D-2F).

### Effects of Diel Cycle on Cultures

**Fig. 3:**
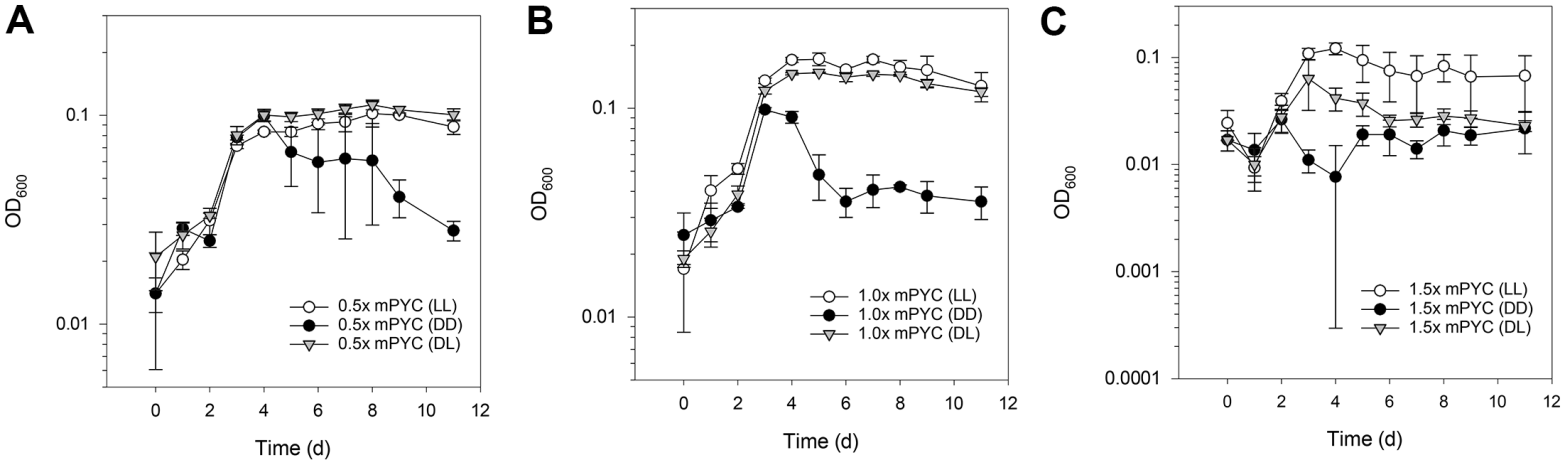
Effect of diurnal diel cycle on IMCC1322 cultures. DL cultures in both 0.5x mPYC and 1x mPYC showed similar growth curves (**A-B**), and 1x mPYC in DL culture also outdid the DD culture after 4 days post-inoculum (**A-B**). In the 1.5x mPYC condition, the DL culture appeared to have a repressed growth curve, falling between the LL curve and the DD curve after 4 days post-inoculum (**C**). Initial inoculum density was 10% or 1/10 volume of the cultures.

In addition to LL and DD conditions, DL cultures were grown using 10% (1/10) inoculum (Fig. 3). DL condition in 0.5x mPYC and 1x mPYC exhibited similar curves to that of LL condition at 4 days post-inoculum (Fig. 3A and 3B). In 1.5x mPYC condition, DL culture seemed to have repressed the growth curve under the LL curve and over the DD curve at 4 days post-inoculum (Fig. 3C).

### Changing Light Regimes in Stationary/Death Phases

**Fig. 4:**
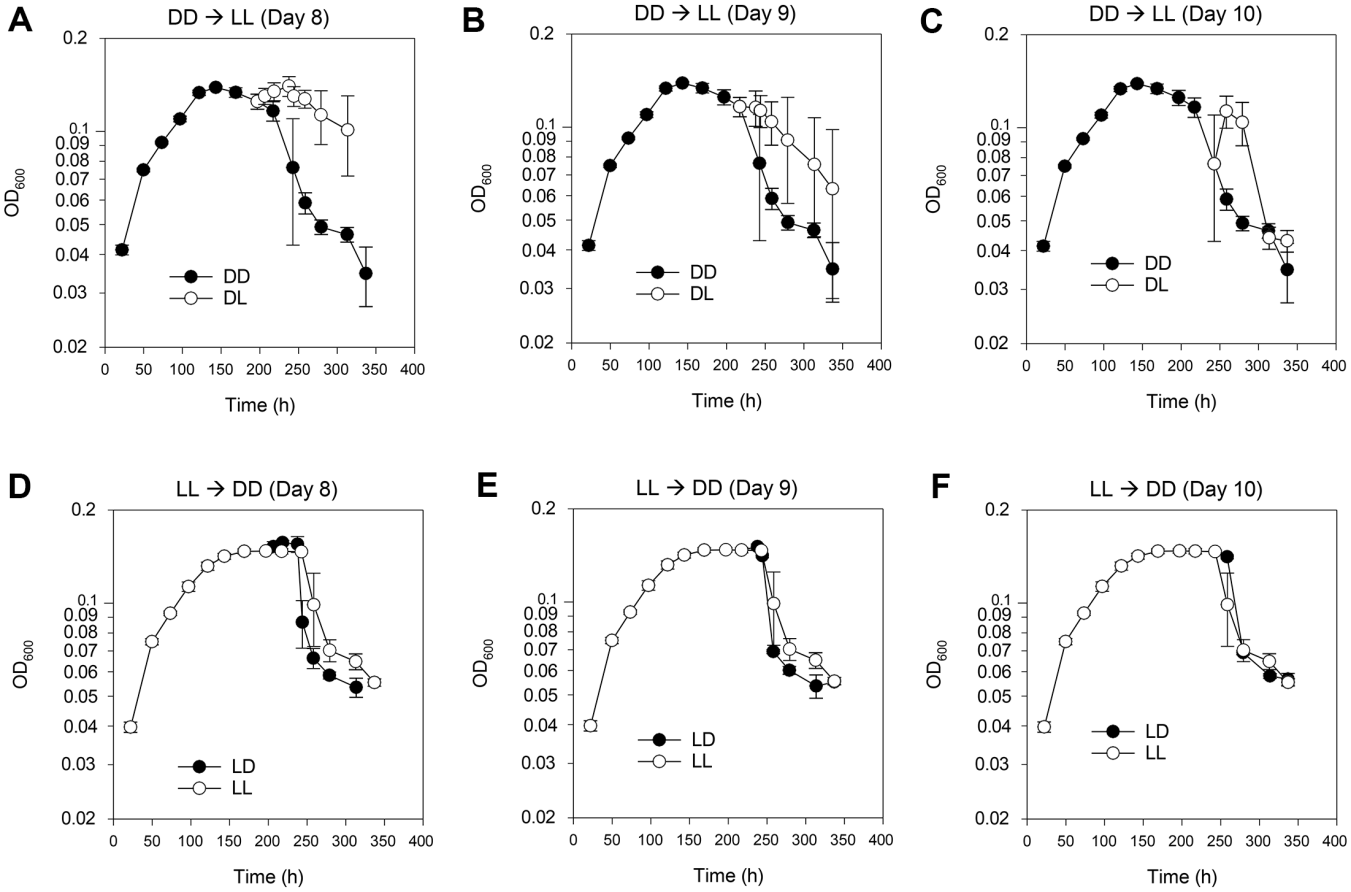
Effect of switching lights on or off in IMCC1322 cultures during the stationary or death phase. Over 8 days post-inoculum, the periods for DD and LL cultures were extended; taking samples from the DD or LL culture on days 8, 9, and 10 and then transferring them to the opposite LL or DD conditions, respectively. Initial inoculum density was 2% or 1/50 of the 1x mPYC culture volumes. When we changed the light regime from DD to LL (DL), the OD_600_ values increased compared to the remaining DD cultures (**A-C**). However, LL culture and the transition from LL to DD (LD) showed a decrease in OD_600_ values. The aging curves of LL cultures definitely began from 9 days post-inoculum (**D-F**).

When the phases of DD and LL cultures were extended to over 8 days, aliquots of DD culture on days 8, 9, and 10 were transferred to LL conditions, and vice versa for LL cultures (Fig. 4). DD cultures whose light regimes were alternated from DD to LL (DL) showed an increase in OD_600_ values compared to the values for the remaining DD cultures (Fig. 4A-4C). However, OD_600_ values for LL culture were not maintained during the transition from LL to DD (LD) (Fig. 4D-4F). The aftermath of aging in LL culture began 10 days post-inoculum (Fig. 4F).

### ATP Levels of Cells Under Changing Light Regimes in Stationary/Death Phases

**Fig. 5:**
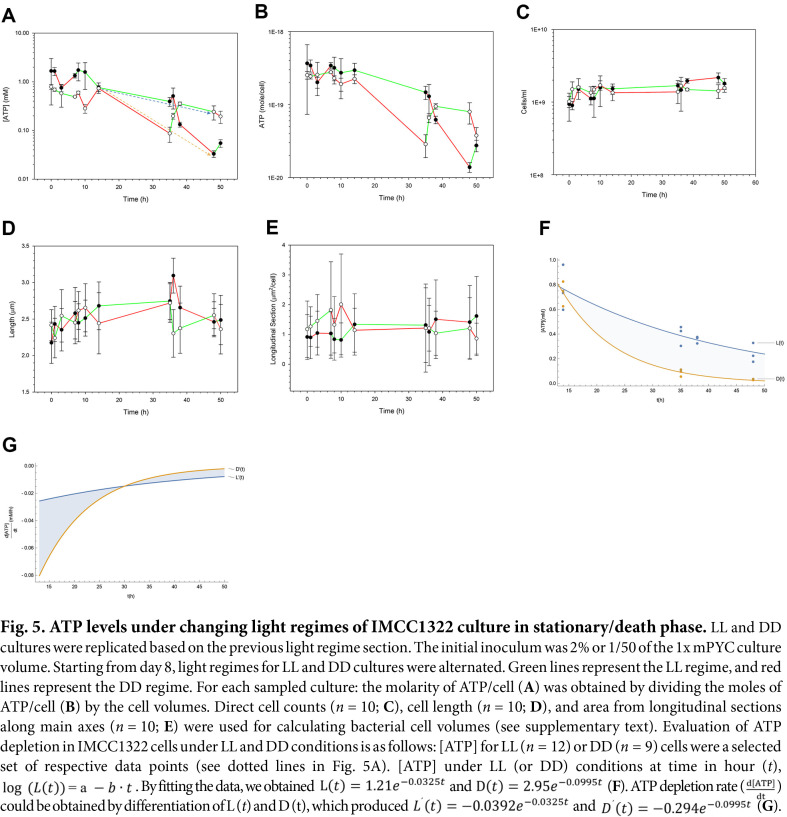
Fig. 5

LL and DD cultures were replicated following the previously described light regime design. Initial inoculum was 2% or 1/50 volume of the culture. Alternation of light regimes for LL and DD cultures were performed from 8-day post-inoculum; green lines for LL regime and red lines for DD regime were as shown in Fig. 5. LL cultures were alternated with DD conditions in the sequence LL, DD, LL, DD, and so on, similar to the alternation for DD cultures in DD, LL, DD, LL, and so on.

Each sampled culture molarity of ATP was determined (Fig. 5A) using moles of ATP/cell (Fig. 5B) divided by cell volumes (see supplementary text and Fig. S1). Direct cell count (*n* = 10; Fig. 5C) as well as bacterial cell volumes calculated by cell length (*n* = 10; Fig. 5D) and area of longitudinal section from major axes (*n* = 10; Fig. 5E) were obtained by ImageJ. Using cell length (L) and area (A), bacterial cell volumes were calculated (see supplementary text and Fig. S1).

The concentration of ATP/cell in alternating DD and LL light conditions was measured, and data points were selected to estimate [ATP] over time (Fig. 5F). For ATP depletion rate in IMCC1322 culture under LL and DD conditions, we fit the data to a model of exponential decay (Fig. 5G).

For [ATP] (mM) under LL condition at time in hour (t), ATP level L (t) could be expressed as in Eq. (1):

$$\log\left(L\left(t\right)\right)=a-b\cdot t.$$ (1)

By fitting the data, we obtain the following Eq. (2):

$$L\left(t\right)=1.21e^{-0.0325t}.$$ (2)

For [ATP] under DD condition at time in hour (t), we also obtain D (t) as the following Eq. (3):

$$D\left(t\right)=2.95e^{-0.0995t}.$$ (3)

ATP depletion rate ($\frac{d\left[ATP\right]}{dt}$) (mM/h) could be obtained by differentiation of L (*t*) and D (*t*):

$$L'\left(t\right)=-0.0392e^{-0.0325t}.$$ (4)

$$D'\left(t\right)=-0.294e^{-0.0995t}.$$ (5)

Integrating L'(*t*) – D'(*t*) from 14 to 48 (h), we get [ATP] difference, 0.196 mM:

$$\int_{14h}^{48h}\left(L'\left[t\right]-D'\left[t\right]\right)dt=0.196mM.$$ (6)

### Measurement of δ^13^-C or δ^15^-N for LL and DD Cultures

Grown with addition of 2.5 g of ^13^C-NaHCO_3_, the LL and DD cultures (~300 ml) were harvested at late log phase and showed δ^13^-C values that were not significantly higher for LL cultures (+^13^C-NaHCO_3_) than δ^13^-C values for DD culture (+^13^C-NaHCO_3_) (*n* = 3; Student’s *t*-test *p* < 0.01) (see Table S1 and Fig. S2). With urea as the control, δ^13^-N values of both conditions showed no significant differences.

### Amino Acids Profiling and Amino Acid Biosynthesis in *Candidatus* Puniceispirillum marinum IMCC1322

The relative amino acid profiles in the protein synthesis of strain IMCC1322 were proposed from RNAseq data from our recent study [24], and they were drawn as a spider plot in supplementary text (Fig. S3).

In Table S2, the amino acids and ions added into 1x mPYC were calculated according to their description in the BD Difco Manual of Microbiological Media [28]. The available growth tests of IMCC1322 cultures, indicated by raised turbidity in 1x mPYC augmented with specific amino acids, were recorded as positive (+), weakly positive (w), or negative (-) based on our previous study [7] (Table S2).

The amino acid biosynthetic capability of strain IMCC1322, as obtained from KEGG and shown in Fig. S4, is detailed in Table S3, which illustrates the energetic cost of canonical amino acid synthesis in IMCC1322. The results from Tables S2 and S3 were combined to create the anaplerotic diagram of gluconeogenesis, the glyoxylate bypass, and the TCA cycle, as shown in Fig. S5.

## Discussion

Our previous study showed little potential for improvement in bacterial biomass, as measured by total cell count and cell absorbance (OD_600_), in LL cultures compared to DD cultures for strain IMCC1322 [7]. DD cultures slightly outperformed LL cultures in nutrient-depleted conditions, except for the culture inhibited by headspace carbon monoxide [7]. However, PRp was observed in nutrient-replete cultures of IMCC1322 in this study (Fig. 1), and this phenomenon was boosted even more by the higher cell densities for inoculation (Fig. 2). Unlike our previous culture study [7] and other reports (Table 1), *Candidatus* Puniceispirillum marinum IMCC1322 showed PRp based on biomass measured in bacterial absorbance (λ = 600 nm) (Figs. 1F and 2C). Such results are highly encouraging for those who have been interested in biological carbon cycle in the ocean where a considerable amount of particulate carbon is produced via oceanic photosynthetic processes, including PRp, oxygenic, and anoxygenic aerobic photosynthesis [29, 30]. PRp of IMCC1322 was observed by increased OD_600_ values at 4 days post-inoculum under LL condition (Figs. 1 and 2), which might have been due to the quenching of quorum-sensing (QS) signals by the augmentation of organic carbon and nitrogen from amino acids in the basal medium (mPYC), and/or by the elevation of cell density. This observation contradicts the common understanding that oligotrophs cannot overcome nutrient limitations [31-34]. Under light regimes (diel cycle and LL), cultures were affected by the light exposure when appropriate conditions were fulfilled (Fig. 3). Using stationary/death phases of culture, alternating light and dark regimes were checked (Fig. 4) and ATP levels were measured for the stationary/death phases of the cultures (Fig. 5). The following sections provide a more detailed discussion of PRp in strain IMCC1322 cultures, offering rationales for how a nutrient-enriched medium and cell density influence PRp, based on previous research [24]. Increased ATP concentrations under LL conditions are discussed in detail in the following sections (see Fig. 5A, 5B, and 5F below). Other PRp in both *Dokdonia* sp. MED134 and *Vibrio* sp. AND4 were also determined using changes in biomass; however, not all cellular ATP levels of the cultures in the studies were tracked (Table 1).

### Amino Acid Utilization and Light-Driven ATP Synthesis in Strain IMCC1322

In mPYC medium, all the canonical amino acids for IMCC1322 to feed on were present, and some of them were confirmed to promote growth in mPYC cultures according to a previous report [7] (Table S2). Amino acids in mPYC could be readily utilized for protein translation or in the anaplerotic TCA cycle [35] for pyruvate formation, from which phosphoenolpyruvate would be produced for glucose and fatty acid syntheses, respectively (Fig. S5). When we adopted KEGG pathways (Fig. S4), *de novo* syntheses of histidine, proline, phenylalanine, tyrosine, and tryptophan were impaired and auxotrophies of these amino acids were expected in strain IMCC1322, whereas other amino acids could be synthesized *de novo* or could be fully metabolized in an anaplerotic manner via the TCA cycle (Fig. S5 and Table S3). Arginine, lysine, and BCAAs (branched-chain amino acids; valine, leucine, and isoleucine) could be synthesized *de novo* by the IMCC1322 genome, but degradative reactions for these amino acids may not be feasible (Table S3 and Fig. S4). Alanine, glycine, threonine, cysteine, and serine could be metabolized into 3-phosphoglycerate or pyruvate. Asparagine and aspartate could enter into the TCA cycle via oxaloacetate, glutamate, and glutamine into the TCA cycle via 2-oxoglutarate. Methionine and threonine could be utilized through succinyl-CoA in the TCA cycle (Fig. S5). Not all amino acids in mPYC were the same in terms of ATP expenditure and the biosynthetic roles of precursor metabolites (Table S3). Also, more energy may be required for the biosynthesis of amino acids like aromatic amino acids, histidine, and BCAA than others [36]. However, only aromatic amino acids and histidine need to be added to the medium, without concern for the extra cost from the biosynthetic burden. Except for proline, arginine, lysine, and BCAAs are less expensive amino acids and can be synthesized *de novo* (Table S3). Other amino acids were of a cheaper variety and could serve as economical intermediates into the TCA cycle for anaplerotic reactions, as well as for ATP production (Fig. S5). When there is a high level of amino acids for which the bacteria are auxotrophic, and high ATP-consuming amino acids like BCAAs in the culture of strain IMCC1322, the ATP requirements would be alleviated in the synthesis of expensive amino acids. For example, “*salvage* cultures” in 2.5x mPYC would thrive better than “*de novo* cultures” in 1x mPYC, as light-driven ATP would promote fatty acid biosynthesis and gluconeogenesis using pyruvate from residual amino acids, with a lower energy cost for easier anabolism (Fig. S5 and Table S3). In addition, light-driven ATP under LL conditions (Fig. 1F) would feed forward LL cultures to increase the levels of ATP (GTP), fatty acids, and hexose pool into protein translation, cell membrane, and cell wall respectively, according to the previous transcriptome study [24]. Therefore, the light-enhanced biomass or cellular volume would increase. But what would happen under nutrient-depleted conditions, where BCAA might be in low or limiting concentrations?Regardless of light regimes, one would expect that ATP (Fig. 1A-1D) may not be sufficient to support *de novo* synthesis of expensive amino acids like BCAA, provided auxotrophic amino acids and others are absorbed by ABC transporters (ATP-binding cassette transporters).

### Role and Impact of BCAAs in Strain IMCC1322 Metabolism

BCAAs are indispensable amino acids crucial for bacterial protein synthesis and cellular metabolism [37-39]. Valine, leucine and isoleucine show the highest hydrophobicity and BCAAs are the major constituents of transmembrane regions of membrane proteins [40]. The biosynthesis pathway for BCAAs is the target for herbicide development, similar to the pathways for glutamine and aromatic amino acids [40, 41]. In this regard, a multiplicity of ABC transporters for BCAAs and acetolactate synthases or acetohydroxy acid synthases (AHAS) in strain IMCC1322 indicates that BCAAs were inarguably important in the metabolism of isoleucine and valine in the stationary phase of the cultures, regardless of the light conditions [24]. ABC transporters for BCAA were major QS genes downregulated under LL condition in stationary phases according to a previous transcriptome of strain IMCC1322 [24]. The effect of BCAA biosynthesis and transaminase reaction of BCAA metabolism of IMCC1322 should be considered from various perspectives. The mPYC medium contained 147 μM, 210 μM, and 316 μM of threonine, isoleucine, and valine, all in free forms, respectively (Table S2). Therefore, mPYC had a valine-to-isoleucine ratio (1.50) similar to those of proteome (1.14~1.22), that we could calculate using both the expected amino acids composition of IMCC1322 (Fig. S3) and the previous transcriptome data [24]. Assuming that cytosolic valine as well as extracellular valine in mPYC are expected in sufficient concentrations over those of isoleucine, this would exert feedback inhibitions on AHAS, and stringent responses are prone to being induced regardless of the presence of other amino acids, as shown in other bacterial systems [42-44]. We speculate that premature consumption of isoleucine by bacteria in lag phase stimulates threonine/serine deamination to derive 2-oxobutanoate (2OB) from threonine. Subsequently, 2OB is condensed with pyruvate to form 2-aceto-2-hydroxybutanoate or two pyruvates, to form 2-aceto-2-hydroxypentanoate by AHAS, which would be throttled by valine [45, 46].

In the IMCC1322 genome, except for the activity of transaminase, the degradation of BCAAs is impaired (Table S3). The transaminase products of BCAAs include 2-hydroxy acids, such as 2-hydroxyisocaproic acid (HICA) from leucine, and may have some deleterious effect on IMCC1322 membranes when metabolized. HICA significantly inhibits bacterial growth by disrupting cell membranes, leading to depolarization, rupture, and ultimately, cell death [47]. Such membrane disruption affects vital metabolic processes like DNA replication, TCA cycle, and oxidative phosphorylation, ultimately inhibiting energy metabolism [48]. BCAA biosynthesis can also produce 2-hydroxy acids in strain IMCC1322. Acetohydroxy acids may be synthesized by AHAS from pyruvates and 2OBs; (S)-2-hydroxy-2-methyl-3-oxobutanoate (acetolactate) from 2 pyruvates and (S)-2-aceto-2-hydroxybutanoate (acetohydroxybutyrate) from pyruvate and 2OB. These 2-hydroxy acids would negatively affect the robustness of the cellular membranes and other cellular processes, as in the case of HICA and acetoin [48, 49].

When we checked the underground metabolism of isoleucine biosynthesis according to a previous study on *E. coli* [45], we found that strain IMCC1322 had canonical threonine cleavage for 2OB formation as well as methionine/homocysteine pathways (Fig. S6). The citramalate pathway was impaired, whereas glutamate mutase pathway, propionyl-CoA reductive carboxylation, and pyruvate-formate lyase were missing in IMCC1322 (Fig. S6). However, the levels of 2-hydroxy acids might be negligible since the activity of acetolactate synthase (AHAS) in IMCC1322 might not be as high as it is in lactic acid bacteria and in plant species [47, 49, 50].

### Impact of Light Regimes and Quorum Sensing on Growth and Metabolism in Strain IMCC1322

Increasing inoculum density induced light-enhanced growths in strain IMCC1322, as if the SAR116 bacterium is a copiotroph (Fig. 2). This appeared to contradict the abovementioned nutrient repletion effect on biomass or OD_600_ (Fig. 1F) since higher cell density meant fewer available amino acids remaining in the medium. Reduced amino acid levels or nutrient-depletion in the medium would cause IMCC1322 cells to require more ATP to overcome the situation, and to synthesize BCAA as well as other expensive amino acids that it could synthesize from scratch. In this regard, LL cultures would have an advantage of elevated [ATP] over DD cultures, and thus, LL cultures become inevitably more competitive to show nutrient-augmented growth. Thus LL cultures have shown that “having more energy currency (such as ATP) for PR-bearing bacteria like IMCC1322” is akin to “having ‘home advantage’ for a soccer game.” Conversely, having less ATP due to darkness is like experiencing an ‘away disadvantage’ or ‘lack of local fan support.’ Such light regimes for controlling biomass resulted in the LL, DD, and DL curves shown in Fig. 3. Growth curves similar to those shown in Fig. 3B were assessed to perform a transcriptomic analysis on DD, LL, and diel-cycled cultures of IMCC1322 in a recent study of ours [24].

Additionally, equipped with a light-sensitive photoreceptor (PR), the bacterium’s ATP charge also responds to the light regime in its environment [51]. This scenario appears to be well-suited for studying strain IMCC1322 in our research, as its responses were shown in Fig. 1 and Fig. 2. However, exposure to light may activate the bacterium’s quorum-quenching mechanism, thereby disrupting QS communication and altering behavior [52]. QS involves the auto-inducers or QS molecules that accumulate in the medium on a cell population-dependent manner [53]. When the concentration of autoinducers reaches a certain threshold, specific genes are activated, leading to coordinated responses within the bacterial communities, but environmental factors like nutrient level, pH, and antibiotic stress can also affect QS [54]. When a marine bacterium encounters a nutrient-rich environment and the cell density in the surrounding water increases, its QS mechanism is triggered. QS serves as a communication system that allows bacteria to coordinate behavior based on population density [52]. If the bacterium elongates in response to light, such morphological change—driven by the presence of light [7]—represents a fascinating adaptation. Such adaptations may allow the bacterium to optimize energy utilization and enhance survival in the dynamic marine environment [52]. Marine bacteria, with their intricate communication systems and light-driven behaviors, may play a pivotal role in marine biogeochemical cycles, as proposed in previous studies (Sabehi *et al*., 2005; Urvoy *et al*., 2022). They are not mere, isolated entities; instead, they engage in numerous cell-to-cell interactions significantly contributing to marine ecosystem biodiversity and functioning [29, 30, 52]. However, in our study, it is best that we refrain from drawing hasty conclusions regarding the direct impact of light on quorum quenching. Instead, having a better understanding of cell population dynamics and nutrient demand is essential for making meaningful comparisons between cultures under LL and DD conditions (Figs. 1 and 2). This underscores the importance of understanding SAR116 bacteria when exploring and appreciating the complexity of life and the biogeochemical cycles in the ocean [51, 52]. In addition to cell density, nutrient repletion may also affect QS, as shown in studies of BCAAs in other bacterium or yeasts [55-57].

### Proton Motive Force Management and Membrane Adaptations in Strain IMCC1322

Tradition holds that biomass production (anabolism) increases along with the amount of ATP obtainable from energy-generating pathways (catabolism). However, this was denied empirically in a series of culture studies on the maintenance functions showing that ATP production is not directly related to growth [25]. Strain IMCC1322 in oligotrophic milieus would suffer from excessive proton motive force (PMF) generated by PR under LL conditions. Some bacteria would use overflow mechanisms to secrete organic matter or adopt less efficient ATP-generating catabolic paths [25]. Moreover, futile cycles of potassium, ammonia, and protons exist across the plasma membranes to prepare the bacterial cells for future growth or protection from potential toxic metabolites [25]. IMCC1322 in nutrient-depleted condition had no choice other than to engage in proton spills or futile proton cycles. When there are replete pools of nutrients like amino acids in the basal mPYC (Table S2), excess PMF under LL condition would be generating more ATP by securing pH homeostasis around the plasma membrane. Tolerance of acid stress is important for any bacterium, since excess protons would directly damage the outermost part of the cytoplasmic membrane [58, 59]. The periplasmic spaces of PR-bearing microbes and actively growing heterotrophs may not differ considerably from those of acidophiles or probiotic bacteria in gastric juices. *Acidithiobacillus ferrooxidans* has been known to express the outer membrane porin in response to acidic environment, attempting to control the influx of protons into the periplasm [58]. However, the peak expression of porin genes in IMCC1322 [24] may not be related to the repression of proton influx from the medium, as in the case of *A. ferrooxidans* [58]. Thus, there is a probability that the PR-pumped protons and amino acids are contained between the outer membrane porin channels and plasma membranes. For industrial and probiotic microbes, mitigating membrane damage is of primary concern, along with the intracellular regulation of metabolism and macromolecular repair [58]. Overflowed metabolites and amino acids in culture media would neutralize protons around cell membranes as well as PMF-dependent proton pumps [58]. Moreover, bacteria are able to alter the biophysical aspects of phospholipids by introducing *cis* double bonds, *cis*-to-*trans* isomerization, and *cis*-to-cyclopropane rings, which allows the microbes to thrive in diverse physical environments [60]. Thus, bacteria adapt to the fluidity of cell membranes by adjusting the composition of fatty acids to survive under low pH [58]. Bacteria modify their membrane fatty acids to protect against low pH by increasing unsaturation, converting *cis*-to-*trans* unsaturated fatty acids, incorporating branched and cyclopropane acyl chains, and lengthening the fatty acid chains to reduce acid damage [58]. Cyclopropanation in bacteria is known as a post-synthetic modification of phospholipids, where cyclopropane fatty acids function to cope with acidity, pollutants, and desiccation during freeze-drying [48, 61-64].

### Fatty Acid Composition and Membrane Adaptations in Strain IMCC1322

According to the fatty acid methyl ester profile [7], IMCC1322 comprised 92% unsaturated cellular fatty acids in the form of 1.0% polyunsaturated, and 0.66% cyclopropane acyl moieties. Unsaturated fatty acid chains have double bonds, which prevent the fatty acid chains from packing tightly together, thereby increasing membrane fluidity (permeability) and decreasing membrane resistance [65]. This is contrary to the previous idea that an increased unsaturation ratio enhances bacterial survival under low pH [58], because decrease in membrane fluidity is accompanied by increased saturated fatty acids and decreased unsaturated fatty acids, according to the pH stress response in *A. ferrooxidans* [66]. Such a discrepancy may be due to the bacterial cell wall structures, since the unsaturation in cytoplasmic membrane seemed to be exemplified in the acid tolerance in species from Firmicutes [58]. Bacterial strains like IMCC1322 have a periplasm where they maintain their pH relatively efficiently. Unlike unsaturated fatty acids, which increase membrane fluidity, cyclopropane fatty acids make the membrane more rigid and less permeable [67, 68], which may be acceptable for any bacterial taxa. Unsaturated fatty acids and cyclopropane fatty acids in the plasma membrane of IMCC1322 would antagonize each other, and proton leaking would then be expected due to the high ratio of unsaturated fatty acyl groups in the cellular membrane (93%). Fluidity and permeability of the membrane would cause periplasmic protons to flow back into the cytosol, making the uncoupling of PMF and ATP synthesis inevitable. Such an energy-spilling reaction or futile proton cycle may relieve light-driven pH stress in IMCC1322, without the need for additional uncoupling protein biosynthesis or ATP consumption by F_0_F_1_-ATP synthase.

Acid habituation of *E. coli* and lactic acid bacteria has been reported in relation to the cyclopropanation of fatty acids [58, 68, 69], and that could also be the situation for IMCC1322 and other PR-bearing marine microorganisms, where excess PMF could lead to membrane damage due to intense photon bombardments. Cyclopropane-fatty-acyl-phospholipid synthase (*cfa*; SAR116_0580/SAR116_0655; [EC:2.1.1.79]) in IMCC1322 may converts the *cis* double bonds of unsaturated fatty acyl chains in phospholipid bilayers to cyclopropane rings using S-adenosyl-L-methionine [61, 68]. Genes for cyclopropanation of fatty acids were also encountered in strains HTCC1062 and ISCC53, and *V. campbellii* BAA-1116 among the PR-bearing bacterial strains in this study (data not shown).

Bacteria dissipate energy in futile cycles of ammonium or potassium ions through cell membranes, but proton cycles due to membrane leakage may occur regardless of cellular catabolic and/or anabolic controls [25]. In this regard, strain IMCC1322 may achieve pH homeostasis using unsaturated fatty acyl groups in its cell membrane. Additionally, cyclopropane fatty acid fine-tunes the membrane rigidity, helping IMCC1322 survive in detrimental conditions, such as low pH, temperature, or the presence of toxic compounds, as reviewed in a previous publication [68].

### Oxidative Phosphorylation and Proton Motive Force Management in Strain IMCC1322

Canonical oxidative phosphorylation occurs in IMCC1322 by harnessing the electron transport chains from Complex I to Complex IV to pump protons across the cellular membrane, where F_0_F_1_-ATP synthase is powered to produce ATP (Table 2). In *E. coli*, there are two different respiratory pathways involving different NADH dehydrogenases (NDH-1 and NDH-2) and terminal cytochromes (o and d) [70]. However, strains IMCC1322, HTCC1062, and ISCC53 only contained Complex I as NDH-1 (electrogenic; proton pumping) (Table 2). Bacterial type II NADH:quinone oxidoreductase (NDH-2) has a smaller, simpler, and highly exergonic molecular structure compared to NADH:quinone oxidoreductase (NDH-1), which is not present in mammals and other species [71]. PR-bearing bacteria equipped with Na^+^-NQR (Na^+^-*trans*locating NADH:ubiquinone oxidoreductase) and/or NDH-2 (non-electrogenic) may be able to utilize the light-driven PMF in more flexible and efficient ways, unlike other strains, including IMCC1322, HTCC1062, and ISCC53 (Table 2; see supplementary text for details). *Dokdonia* sp. MED134 has been shown to raise Na^+^-NQR messages that respond to light-drive proton gradients [19]. However, strains like IMCC1322 must inevitably tolerate excessive PMF by ATP-dependent proton pumping, protonation of organic compounds like amino acids and amines, and modifying proton permeability of plasma membranes (see above sections). For oligotrophic bacteria (IMCC1322, HTCC1062, and ISCC53) periplasmic PMF neutralization may not occur primarily through the overflow of metabolites or excess amino acids into the periplasm, though there are proton-dependent sodium antiporters and/or ammonium transporters. Instead, proton influx would be pumped out by consuming ATP and/or dissipation of PMF due to unsaturation and post-synthetic modification of the fatty acid moiety (for unsaturation ratio and cyclopropanation, see above). If the TCA cycle is fully operating in strain IMCC1322, spurring of TCA cycle genes under LL conditions is not necessary (Fig. S7). Aging cultures may rely only on endogenous organic carbon pools and the canonical oxidative phosphorylation by NDH-1. Nonetheless, TCA cycle genes would be overexpressed in LL conditions compared to DD conditions [24], and LL light regime in cultures of strain IMCC1322 would shift its NDH-1 into non-electrogenic modes (Fig. S7). This changes the $\frac{ATP}{NADPH}$ ratio, retarding electron transfer and ATP synthesis, as well as repression of NADH from the TCA cycle (Fig. S7). The redox imbalance is alleviated by the bifurcated expression of isocitrate dehydrogenase; IDH1 (isocitrate dehydrogenase 1) for NADPH and IDH3 (isocitrate dehydrogenase for NADH). More NADH would be readily converted into NADPH by NAD (P)^+^-*trans*hydrogenase (Fig. S7). Transhydrogenases in *E. coli* exist in two isoenzymatic forms: one soluble form and the other membrane-bound form [72]. Likewise, membrane-bound proton-*trans*locating NAD (P)+ transhydrogenase in IMCC1322 (Fig. S7; supplementary text) would link hydrogen from NADH to NADPH using PMF. As long as PR is turned on, the TCA cycle is not like a currency exchange (ATP) from acetyl-CoA to oxidative phosphorylation, but like a withdrawal counter for reducing power (NADPH). The resulting anaplerotic pyruvate is driven back up to phosphoenolpyruvate by the key enzyme pyruvate phosphate dikinase, whose function is favored in SAR11 as previously argued [31, 73]. This differs from *Dokdonia* sp. MED134 in that phosphoenolpyruvate is formed by phosphoenolpyruvate carboxykinase and/or pyruvate carboxylase [13].

### Anaplerotic Role of TCA Cycle in Strain IMCC1322

Starch (~0.5 g/l), glucose (~2.76 mM), and then lastly pyruvate (~ 2.65 mM) was omitted from the R2A formula (= mPYC) individually, as these substrates affected the culture of strain IMCC1322 [7]. IMCC1322 is missing 6-phosphofructokinase [EC:2.7.1.11], ADP-dependent phosphofructokinase/glucokinase [EC:2.7.1.146], and diphosphate-dependent phosphofructokinase [EC:2.7.1.90] in Embden-Meyerhof pathway (EMP). Thus, glycolysis from glucose to pyruvate is blocked in IMCC1322, whereas FBP (fructose-1,6-bisphosphatase I [EC:3.1.3.11]) in IMCC1322 (SAR116_1413) may function in a gluconeogenic manner by consuming fructose-1,6-bisphosphatase in the EMP pathway as well as in the pentose phosphate pathway. Not glucose but gluconate supported the growth of IMCC1322 [7], which secured the ED pathway of IMCC1322. Gluconeogenesis, rather than the glycolysis of IMCC1322 is evident due to of the absence of both glucose and pyruvate in mPYC (Table S2). Such gluconeogenic metabolism has also been argued in previous SAR11 studies [31, 73]. Less efficient in ATP generation, the ED pathway offers a selective advantage during periods of rapid growth acceleration owing to its strong thermodynamic driving force, streamlined glucose import, and ability to promote rapid adaptation to intermittent nutrient supply [3, 74]. Through the evolution, IMCC1322 might have abandoned glycolysis, with FBP as the last resort for its gluconeogenic reaction, as discussed in SAR11 studies [31, 73]. Although extracellular glucose amendment does not affect IMCC1322, the ED pathway genes are involved in gluconate utilization and the intracellular hexose pool [7]. Based on proton/gluconate transporters, an evolutionary advantage of the ED pathway over the EMP pathway has been suggested according to previous arguments [3, 74], and this is understandable for IMCC1322 and some SAR11 strains. When 4g/L of glucose, glycerol, and acetate were fed into *E. coli* cultures, cellular ATP was the lowest for an acetate-fed culture [75]; however, the lowest ATP level of acetate-fed *E. coli* may have been due to the gluconeogenesis of the hexose-phosphate pool for peptidoglycan and other related metabolic pathways. Glycerol is a more competent carbon and energy source for amino acids and protein translation in *E. coli* [76], but glycolysis and glycerol metabolism may be fed forward into the toxic methylglyoxal shunt [25, 75]. Consequently, oceanic niche-specific bacteria such as IMCC1322 and SAR11, may not rely on glycolysis, even if a hexose exists in their environment, and they favor the anaplerotic TCA cycle to make up for other intermediates [35].

### ATP Levels and Light-Regime Effects on Strain IMCC1322 After the Log Phase

ATP levels in strain IMCC1322 under alternating LL/DD conditions were measured 8 days post-inoculation (Fig. 4). [ATP] ranged from 0.0331 to 1.74 mM (Fig. 5A), corresponding to 13.9 to 367 zeptomoles of ATP/cell (Table 1 and Fig. 5B). Unlike the pulse and chase of photon irradiance in *Candidatus* Pelagibacter ubique HTCC1062 [4] and *Candidatus* Pelagisphaera phototrophica ISCC53 [18], we measured ATP levels in cultures during the stationary and death phases under hourly-to-daily light regime changes (Fig. 5). The ATP concentrations of the other PR-bearing *Vibrio* strains, specifically, *V. campbellii* BAA-1116 and *V. campbellii* CAIM519, which lack PRp, were measured (Table 1). *V. campbellii* BAA-1116 showed an up-shift in [ATP] under light conditions using an ATCC 2034 medium containing 137 mM glycerol, 1 mM L-arginine, and 2.0% casamino acids (10.3 mM free amino acids) [10]. However, *V. campbellii* CAIM519 showed a down-shift in [ATP] regardless of C-limited ASW (2.78 mM maltose / 2 mM NH_4_Cl) and/or N-limited ASW (8.34 mM maltose / 1 mM NH_4_Cl)[11]. Relatively higher nutrient levels in media may result in higher [ATP] in *V. campbellii* strains. Basal ATP levels were dependent on the organic carbon concentrations in the media, but these observations did not always coincide with the PRp, as summarized in Table 1.

Protein translation is a growth-related function, whereas protein turnover occurs during degradation and re-synthesis [25]. Protein biosynthesis is the most energy-consuming process in bacteria, consuming about 50-70%of the ATP pool [25, 77], as argued in a previous report [24]. We checked the IMCC1322 cultures for light-regime shifts (Fig. 4), and subsequently tracked ATP levels from the log phase to the stationary/death phase (Fig. 5). As shown in Fig. 5F, cellular ATP levels in LL culture may well outcompete DD cultures, even if we alternate light and dark regimes according to the experimental design. Although we did not study continuous culture to measure the maintenance energy or endogenous metabolism of strain IMCC1322, differences of ATP levels between light and dark conditions could be inferred. The overall differences in ATP levels between light- and dark-conditioned cells were roughly estimated to be 0.196 mM by integration over 34 h (Fig. 5G; Eq. 6). If we assume strain IMCC1322 is similar to *E. coli* in translational efficiency, 4 ATP equivalents per amino acid polymerization, an average molecular mass of 110 Dalton for any amino acid, 0.5 g of protein/g of cell biomass [25], and excess ATP under light condition (0.196 mM/34 h) (Fig. 5G) would correspond to a protein synthesis ratio of 0.159 mg/l/h or g biomass of 0.317 mg/l/h. Assuming the average bacterial volume of strain IMCC1322 as 3.44 ×10^-16^L/cell (Fig. 5F and 5G), excess ATP value (0.168 zmol/cell/h) could be converted into protein synthesis of 5.45 × 10^-20^ g/cell/h and biomass of 1.09 × 10^-19^ g/cell/h respectively. The rate of protein turnover for *E. coli* in log phase ranges from 0.5 to 2.5%/h, and at stationary phase 5%/h [25]. Strain IMCC1322 had cellular levels of ATP 13.9~367 zmol/cell after the log phase (Table 1). If we assume 50~70% of the ATP pool is directed toward protein biosynthesis [24], cells consume ATP at 6.95~9.73 zmol/cell/h for protein biosynthesis. Meanwhile, 5% protein turnover corresponds to 0.348~0.487 zmol/cell/h of ATP. However, enumerated cellular ATP level of light and dark cultures differed by only 0.168 zmol/cell/h on average for stationary phase (Fig. 5G), and this value is too inconsequential an amount of ATP at minimal 13.9 zmol/cell. A light-driven ATP rate of 0.348 zmol/cell/h was required to compensate for protein turnover in an almost dead cell to keep up with 13.9 zmol ATP/cell. However, the additional PR-driven ATP synthesis rate was 0.168 zmol/cell/h on average, which was less than half the expected value. In some instances, during the late log phase, LL cultures may have advantages over DD cultures because of the sustained elevated ATP synthesis (Fig. 4). As a photoheterotrophic strain, IMCC1322 depends on organic substrates as its carbon and energy sources; however, PR can never be harnessed to generate NAD (P)H for anabolic metabolism. A chloroplast proportionately controls the supply of both ATP and NADPH to improve photosynthetic productivity [78] unlike the PR function of strain IMCC1322, which supplies only ATP. This result was also supported by the negligible differences in the ^13^C-stable isotope values between LL and DD conditions (see Table S1 and Fig. S2). Stable isotope measurements for inorganic carbon incorporation into the bacterial cells of IMCC1322 revealed no significantly higher PR-driven ATP synthesis in LL culture than in DD culture (*n* = 3; Student’s *t*-test *p* < 0.01) (see Table S1 and Fig. S2).

### Impact of Light-Driven ATP Synthesis on Nutrient-Limited Cultures of Strain IMCC1322

The difference in light-driven ATP synthesis did not significantly affect the nutrient-limited cultures (in 1/10 ~ 1 x mPYC) in lag and log phases. Contrary to the promising bacterioplanktonic PRp on the sea surface [1, 2, 79, 80], only a few studies on strains such as IMCC1322, *Dokdonia* sp. MED134/DSW-1, and *Vibrio* sp. ADN4 were meaningful with respect to PRp when the cellular volume or biomass changes were considered (Table 1). Strain IMCC1322 did not exhibit light-enhanced growth [7], possibly due to the nutrient-limited conditions that restricted the utilization of light-driven PMF, similar to the situation observed in stationary phase cultures. Unfavorable acid stress caused by protons accumulated by PR could be mitigated by the protonation of periplasmic metabolites, proton-pumping F_0_F_1_-ATP synthase, and other proton-dependent ionotropic membrane proteins, if they are present [25]. Light-driven ATP would be consumed for the futile proton cycle for pH homeostasis, and ATP would not be shunt into anabolic processes like RNA polymerization and protein translation in nutrient-limited cultures [24]. Accordingly, strain IMCC1322 could alleviate light-driven proton overflow in the periplasm under nutrient limitation, which might explain why DD cultures outcompeted LL condition cultures [7]. However, PR-bearing marine bacterioplanktons have shown visible biomass gains under light conditions if periplasmic factors like amino acids, Na^+^-NQR, and proton-dependent ionophores that may mitigate unwarranted PMF in light-driven PR are present. The most effective mitigation of excessive light-driven PMF might be exemplified by strains with Na^+^-NQR from the genera Dokdonia and *Vibrio* [9, 12, 15, 19] (Table 2). Without Na^+^-NQR and with only a few proton-dependent ion pumps, IMCC1322 relies on amino acids to neutralize light-driven protons in the periplasm. This process negates light-driven ATP synthesis, thereby depleting the intracellular ATP pool from endogenous metabolism.

### Competitive Strategies of Strain IMCC1322 and PR-Bearing Marine Oligotrophs

In this study, strain IMCC1322 from the SAR116 clade exhibited light-enhanced growth under more copiotrophic conditions, which we did not expect. This finding challenges the consensus that oligotrophs cannot overcome nutrient limitations [31-34]. Heterotrophs in both high- and low-nutrient environments express PRs (Table 1), and it has been argued that more studies are needed to determine if their photophysiology varies based on nutrient adaptation [81]. PR-bearing bacteria like SAR11 and SAR116 utilize light-driven proton pumps (PR) to generate ATP, which can be advantageous in nutrient-limited environments [13, 51, 81].

In natural environments, bacteria frequently engage in intense competition for the same energy sources [25]. Although light energy is not of primary importance for photoheterotrophs like SAR11 and SAR116 in the open ocean, these bacteria have adapted to become more potent competitors. When a bacterium is limited by factors other than its energy source, its current energy-spilling behavior might soon become beneficial. In other words, a bacterium might strategically waste more energy than needed to prepare for a more advantageous future [25]. However, solar energy cannot be worn out easily and therefore cannot be finitely wasted, unlike glucose, which could otherwise be utilized by potential competitors. Both SAR11 and SAR116 adopt the ED pathway, which is more thermodynamically favorable. This pathway saves phosphate and avoids the methylglyoxal shunt by repressing EMP enzymes, although the ED pathway is less efficient in ATP production compared to the EMP pathway [25]. If PR-pumped protons exceed the capacity and resistance of cellular membranes, the dielectric effects of phospholipids require consideration. The theoretical maximum electrical potential of 500 mV for bacterial membranes may be scarcely achieved [25], similar to heterotrophs experiencing excessive metabolic activity. To avoid deleterious membrane damage caused by the dielectric effect of elevated PMF, the electrical potential of the membrane is actually controlled below the practically tolerable level of 200 mV in PR-bearing bacteria [25, 82].

To relieve the excess membrane electric potential generated by PR, IMCC1322 might rely on the protonation of amino acids from the nutrient-replete media. This results in light-enhanced biomass or an increased cell volume. Such strategies controlling light-driven PMF (or ATP) may be well optimized in IMCC1322 from SAR116, which is typical for marine oligotrophs like SAR11 and other PR-bearing bacteria. Marine oligotrophs could utilize PR to harness solar energy or not. However, PR drives PMF generation, which depletes the ATP pool and damages the dielectric phospholipids in cell membranes. Despite the risk of fatal energy levels to cell membranes, PR-bearing bacteria have developed strategies to become more potent competitors in the bacterial world. The concept of “energy spilling” as a strategy to outcompete others by wasting energy to position themselves favorably [25] has not been well documented for these types of bacteria.

## Conclusion

Strain IMCC1322, a SAR116-clade bacterium, showed light-stimulated growth under high concentration of amino acids and high initial cell densities. To link the light-responsive physiology to nutrient conditions, we speculate that excess light-driven protons may be mitigated by amino acids absorbed in the periplasm, thus maintaining cell membrane integrity. Some amino acids in the medium may also quench QS and/or the stringent response of strain IMCC1322. However, light-driven ATP synthesis could not aid protein turnover in strain IMCC1322 under nutrient-limited conditions. Most research on PR-bearing bacteria has concentrated on their efficient utilization of light energy. The concept of deliberately wasting energy to gain a competitive advantage has not been a primary focus in these studies [11]. In this study, we demonstrated that the ecological niche of SAR116 and other PR-bearing bacteria with impaired photoheterotrophy might involve energy spilling rather than energy efficiency when utilizing near-finite sunlight dispersed over the open ocean. Future studies should further explore these dynamics to gain deeper insights into the ecological roles and metabolic adaptations of PR-bearing bacteria.

## Supplemental Materials

Supplementary data for this paper are available on-line only at http://jmb.or.kr.



## Figures and Tables

**Table 1:** Proteorhodopsin phototrophy study conducted on marine bacteria.

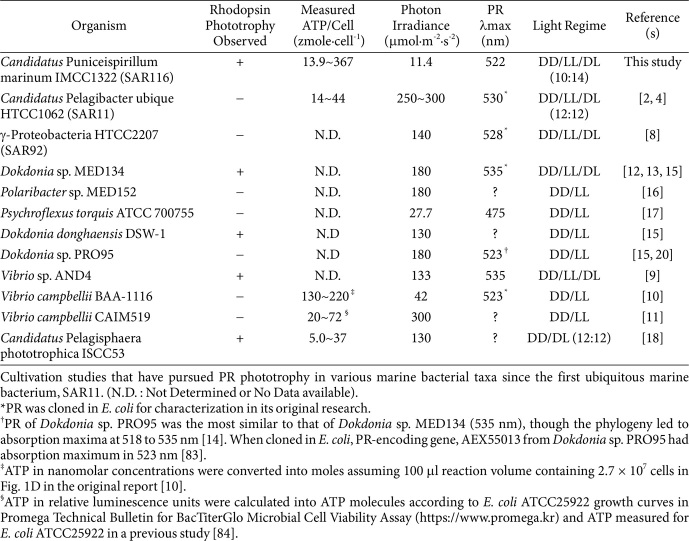

**Table 2:** Oxidative phosphorylation genes in PR-bearing marine bacteria.