# Corrigendum to: Effect of Light Regime on *Candidatus* Puniceispirillum marinum IMCC1322 in Nutrient-Replete Conditions

**DOI:** 10.4014/jmb.2025.3503.C01

**Published:** 2025-02-28

**Authors:** Hyun-Myung Oh, Ji Hyen Lee, Ahyoung Choi, Sung-Hyun Yang, Gyung-Hoon Shin, Sung Gyun Kang, Jang-Cheon Cho, Hak Jun Kim, Kae-Kyoung Kwon

**Affiliations:** 1 Institute of Liberal Arts Education, Pukyong National University, Busan 48547, Republic of Korea; 2 Department of Pediatrics, Ewha Womans University School of Medicine, Seoul 07804, Republic of Korea; 3 Nakdonggang National Institute of Biological Resources, Sangju 37242, Republic of Korea; 4 Korea Institute of Ocean Science and Technology, Busan 49111, Republic of Korea; 5 Hanyang University ERICA, Ansan 15588, Republic of Korea; 6 Division of Biology and Ocean Sciences, Inha University, Incheon 22212, Republic of Korea; 7 Department of Chemistry, Pukyong National University, Busan 48547, Republic of Korea

J. Microbiol. Biotechnol. 2025. 35: e2410034

https://doi.org/10.4014/jmb.2410.10034

This corrigendum is being published to correct the last author’s name and affiliation.

- Kae-Kyoung Kwon should read as Kae Kyoung Kwon.

- The author's affiliation is Korea Institute of Ocean Science and Technology, and the affiliation number should also be corrected to No. 4.

The corrected version of this article is available in the Archive (http://jmb.or.kr)

