# Supplementary material for: Effect of Light Regime on Candidatus Puniceispirillum marinum IMCC1322 in Nutrient-Replete Conditions
Source: J Microbiol Biotechnol. 2024 Nov 27;35:e2410034. doi: 10.4014/jmb.2410.10034 (PMC11813361; doi:10.4014/jmb.2410.10034)
Supplement: Supplementary file 1 [file jmb-35-e2410034-supple.pdf]

J. Microbiol. Biotechnol. 2025. 35: e2410034

https://doi.org/10.4014/jmb.2410.10034

Supplementary Figures

Effect of Light Regime on *Candidatus* Puniceispirillum marinum IMCC1322 in Nutrient-Replete Conditions

Hyun-Myung Oh[1*], Ji Hyen Lee[2*] Ahyoung Choi[3], Sung-Hyun Yang[4], Gyung-Hoon Shin[5], Sung Gyun Kang[4], Jang-Cheon Cho[6], Hak Jun Kim[7,†], and Kae-Kyoung Kwon[5*]

1. Institute of Liberal Arts Education, Pukyong National University, Busan 48547, Republic of Korea
2. Department of Pediatrics, Ewha Womans University School of Medicine, Seoul 07804, Republic of Korea
3. Nakdonggang National Institute of Biological Resources, 137 Donam 2-gil, Sangju 37242, Republic of Korea
4. Korea Institute of Ocean Science and Technology, Busan 49111, Republic of Korea
5. Hanyang University ERICA, 55 Hanyangdaehak-ro, Sangnok-gu, Ansan-si, Gyeonggi-do 15588, Republic of Korea
6. Division of Biology and Ocean Sciences, Inha University, Yong-Hyun-Dong, Nam-Gu, Incheon, 22212, Republic of Korea
7. Department of Chemistry, Pukyong National University, Busan, 48547, Republic of Korea

* These authors equally contributed to this work.

[†] Corresponding authors' contact

Hak Jun Kim : e-mail: kimhj@pknu.ac.kr, phone : +82 51 629 5587

[‡] Corresponding authors' contact

Kae-Kyoung Kwon : e-mail: kkkwon@kordi.re.kr, phone : +82 51 664 3371

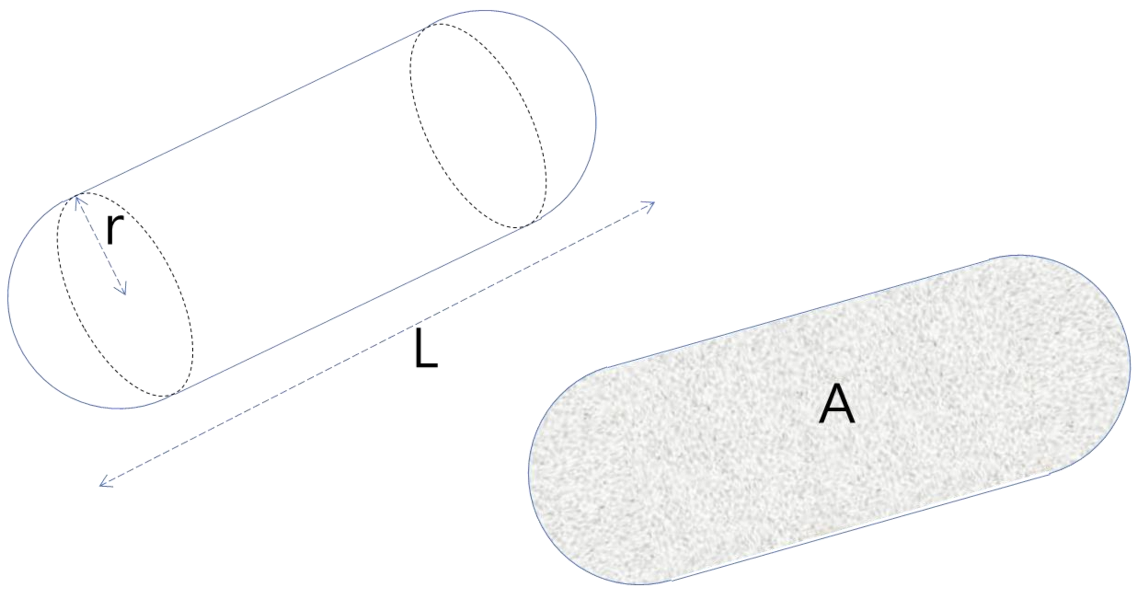

Fig. S1. Geometry of bacterial rod with cellular length L and area of longitudinal section A along the major axis.

Bacterial Volume Calculation Using Microscopic Images of Rods

Figure S1 depicts the geometric model of a bacterial rod used for volume calculation, model ed as a cylinder with hemispherical ends. The length of the cylinder is denoted by L, and the radius shared by both the cylinder and hemispheres is represented by *r*. The longitudinal sec tion of the rod, displayed along the major axis, has area *A*. To calculate the volume *V* of the bacterial rod, the following steps are applied:

The volume of the cylindrical component *C* is given by $C = \pi(L - 2r)r^2$

The volume of the spherical caps *Sv* is twice that of a hemisphere, computed as $Sv = \frac{4\pi r^3}{3}$

Thus, the total rod volume *V* is the sum of the cylindrical and spherical volumes:

$$V = \frac{4\pi r^3}{3} + \pi(L - 2r)r^2$$

This expression simplifies to $V = \frac{1}{3}\pi(3L - 2r)r^2$.

The area of the longitudinal section *A* is calculated using the formula $A = 2(L - 2r)r + \pi r^2$.

The correct radius *r*, which is crucial for these calculations, is derived from *A* and the length *L* of the rod. For the given ranges of *A* (0.05 < *A* < 14) and *L* (0.8 < *L* < 7), *r* can be calculated using the following quadratic formula, where a positive radius is necessary for a physical representation:

$$r = \frac{-L + \sqrt{L^2 + A(-4 + \pi)}}{-4 + \pi}$$

The total volume *V* of the bacterial rod can be calculated upon obtaining *r*. If the computed volume is negative, it is rejected as it does not represent a viable physical object; conversely, a positive volume is accepted. The overall formula used to compute *V* is

$$V = \frac{\left(L + \sqrt{L^2 + A(-4 + \pi)}\right)^2 \pi \left(2\sqrt{L^2 + A(-4 + \pi)} + L(-10 + 3\pi)\right)}{3(-4 + \pi)^3}$$

Here, a positive volume reflects the actual space occupied by the bacterial rod, which is essential for accurate biological analysis.

Solve $A = 2\,(L-2r)r+\pi\, r^2$

$$(\, r \to \frac{-L+\sqrt{L^2+A(-4+\pi)}}{-4+\pi}, r \to -\frac{L+\sqrt{L^2+A(-4+\pi)}}{-4+\pi}\,)$$

If $r = \frac{-L+\sqrt{L^2+A(-4+\pi)}}{-4+\pi}$, and [0.05 <A< 14, 0.8 <L<7] then

$$V = \frac{\left(L + \sqrt{L^2 + A(-4+\pi)}\right)^2 \pi \left(2\sqrt{L^2 + A(-4+\pi)} + L(-10+3\pi)\right)}{3(-4+\pi)^3}$$

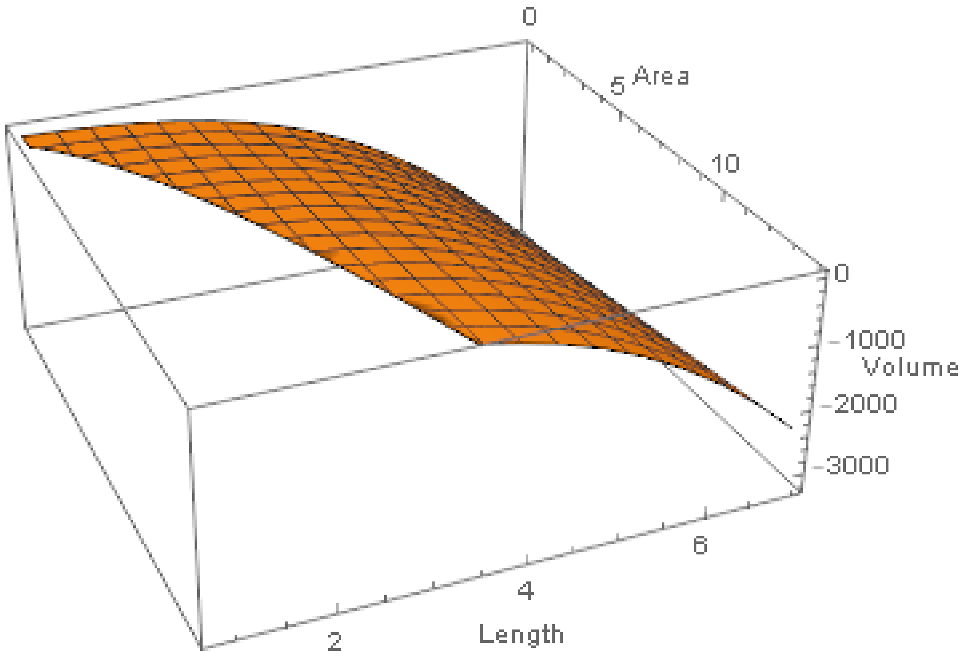

Negative volumes were rejected to ensure that the physical constraints of the bacterial rod geometry were satisfied. This model facilitates the analysis of bacterial size and shape through microscopic images, contributing to a better understanding of bacterial cell structure.

If $r = -\frac{L+\sqrt{L^2+A(-4+\pi)}}{-4+\pi}$, and [0.05 <A< 14, 0.8 <L<7] then

$$V = \frac{\left(L - \sqrt{L^2 + A(-4+\pi)}\right)^2 \pi \left(-2\sqrt{L^2 + A(-4+\pi)} + L(-10+3\pi)\right)}{3(-4+\pi)^3}$$

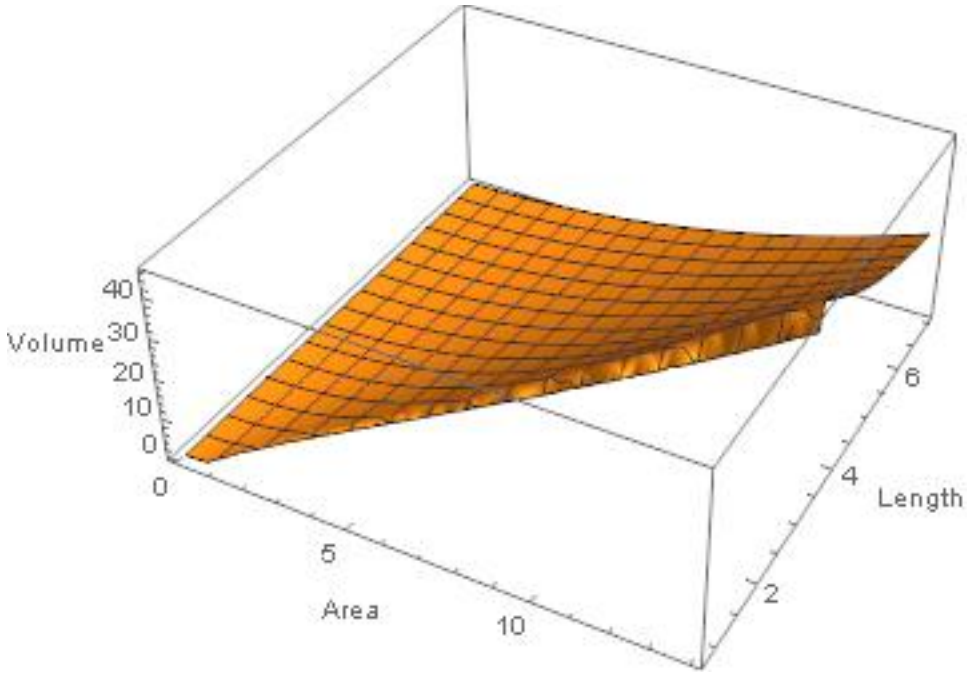

A positive volume value is a prerequisite for validity as it signifies a real object in three-dimen

sional space.

For V > 0 then accept

$$\therefore V = \frac{\left(L - \sqrt{L^2 + A(\pi - 4)}\right)^2 \pi \left(L(3\pi - 10) - 2\sqrt{L^2 + A(\pi - 4)}\right)}{3(\pi - 4)^3} (\because V > 0)$$

Based on above calculations, bacterial cell volumes(V) were enumerated using the cell lengt hs and longitudinal section areas (Fig. 5D and Fig. 5E).

Oxidative Phosphorylation in PR-bearing IMCC1322

Canonical oxidative phosphorylation occurs in IMCC1322 by harnessing the electron transport chains from Complex I to Complex IV to pump protons across cellular me mbrane where F_0F_1-ATP synthase is powered to produce ATP (Table 2). LL conditio n stimulated genes for succinate dehydrogenase/fumarate reductase (Complex II), c ytochrome bc1/ubiquinol cytochrome c reductase (Complex III), cytochrome c/coxAB C (Complex IV), and F-Type ATP synthase genes (Complex V) according to the RN Aseq of IMCC1322 study [1]. Genomic comparison of the bacteria in this study was s ummarized for oxidative phosphorylation (Table 2), and the genomes from other than strains IMCC1322, HTCC1062 , and ISCC53 had canonical oxidative phosphorylatio n system. Other bacterial genomes from HTCC2207, three *Vibrios*, and five Flavoba cteria were checked to be harboring nqrABCDE (Na^+- translocating NADH:ubiquinon e OxR [EC:7.2.1.1]) or Na^+-NQR [2] instead of NADH:quinone oxidoreductase [EC: 7.1.1.2] (Complex I in Table 2), and PR and Na^+-NQR relationship were confirmed in the study[3] . Moreover, *Psychroflexus* torquis ATCC 700755, and *Polaribacter* sp. MED152 had NADH:quinone reductase(RD) (non-electrogenic). Bacterial type II NA DH:quinone oxidoreductase (NDH-2) is an attractive antimicrobial drug target since it has smaller, simpler, and highly exergonic molecular structure than NADH:quinone oxidoreductase (NDH-1), which is not present in mammals and other species [4].

There was shift down of some NDH-1 subunits of Complex I under LL condition stati onary/death phase [1]. By the analogy to a previous report [5] subunits of NADH:qui none OxR (NDH-1) can be grouped into N, P, and Q-modules: NADH-binding, pumpi ng, and Q-binding modules. Moreover, bacterial NDH-1 containing 14 conserved cor e subunits can form the hydrophilic peripheral arm (PA) and the membrane arm (MA)

joined in an L-shape [6, 7]. Subunits of P-module (*nuoA*, *nuoH*, and *nuoJKLMN*) mig ht well belong to hydrophobic MA of NDH-1, and the other core subunits consisted of N-module(*nuoEFG*) and Q-module (*nuoBCD* and *nuoI*). Whereas P-module (*nuoH*, and *NuoJKLMN*) on MA was repressed under LL condition unlike N-module (*nuoEF G*) and Q-module (*nuoBCD* and *nuoI*) under LL condition in stationary/death phase [1]. If NDH-1 were to lose or stop its ability of P-module (becoming nonelectrogenic li ke NDH-2), ATP production may decrease and redox balance of electron carriers ($\frac{NADPH}{NADP^+}$ or $\frac{NADH}{NAD^+}$) may be disturbed. Such down-shift of P-module with blocked electron transfer from NADH would raise $\frac{NADH}{NAD^+}$ to affect redox homeostasis, TCA cycle (or citr ic acid cycle), and ATP synthesis. Isocitrate dehydrogenase 1 (IDH1;SAR116_0090; EC 1.1.1.42) in TCA cycle produce NADPH under LL condition in stationary phase, b ut IMCC1322 had an additional gear for NADH under DD condition in stationary/deat h phase: isocitrate dehydrogenase 3 (IDH3; SAR116_1739~40~41; EC 1.1.1.41) wh ere P-module subunits are favored (Fig. S7).

There has been an argument that the TCA cycle genes were acquired from the mitoc hondrial genome over time because TCA cycle genes are nuclear genes, but TCA cy cle occurs in the mitochondrial matrix [8]. However, bacteria are different in that they exhibit diverse metabolic capabilities with regard to energy production and nutrient ut ilization [9]. So, it is not so difficult for us to came across incomplete TCA cycles in b acteria [10-13]. Although complete TCA cycle would be working properly in strain IM CC1322, there is no reason not to stimulate the TCA under LL in stationary/death ph ase(Fig. S7). Stationary DD culture may rely only on endogenous organic carbon po ol and the canonical oxidative phosphorylation by NDH-1, which makes sense, nonet heless the TCA genes were overexpressed in LL condition, not in DD condition [1].

Moreover, LL light regime in aging cultures of strain IMCC1322 shifted its NDH-1 into non-electrogenic non-functional proton-translocase in turn. This would make $\frac{NADH}{NAD^+}$ ra tio rise in addition to retarded electron transfer and ATP synthesis (Fig. S7). The red ox imbalance would be alleviated by the bifurcated expression of IDH1 for NADPH in stead of IDH3 because NADH in turn would be traded-off by $NAD(P)^+$-transhydrogen ase [14] (Fig. S7). *E. coli* had in two isoenzymic transhydrogenases in soluble forms and membrane-bound forms [15]. Likewise, membrane-bound proton-translocating N AD(P)+ transhydrogenase in IMCC1322 (SAR116_1339/SAR116_1340/SAR116_13 41) would link hydrogen from excess NADH to NADPH using extracellular proton mo tive forces. Consequence of LL condition in aging cultures tells us that when PRp-de pendent ATP production is at constant levels, TCA cycle is not like a currency excha nge (ATP) from acetyl-CoA to oxidative phosphorylation but like a withdrawal counter for reducing power (NAD(P)H). Resulting anaplerotic pyruvate would be driven back up to phosphoenolpyruvate or acetyl-CoA by the key enzyme pyruvate phosphate di kinase whose function would be expected as previously argued in SAR11 studies [1 6, 17]. This is different from *Dokdonia* sp. MED134 in that phosphoenol pyruvate is f ormed by phosphoenolpyruvate carboxykinase and pyruvate carboxylase [18].

The ubiquitous mobile electron carrier ETF and its electron acceptor, ETF-ubiquinon e oxidoreductase (ETF-QO) play essential roles in the β-oxidation of fatty acids, bran ched-chain amino acids, lysine, tryptophan, and N-methyl amino acids (N-methyl-gly cine and N,N-dimethylglycine) in mammalian mitochondria [19]. Such ETF-QO activit y would make sense in strain IMCC1322 (Fig. S7). Electron transfer flavoproteins(ET F-α/ETF-β or FixA/FixB)(SAR116_1869~70) and ETF-QO(or ETFDH)(SAR116_166 2) might well reimburse the quinone pool(Q-QH_2)(Fig. S7) with regard to previous stu

dies [20-22].

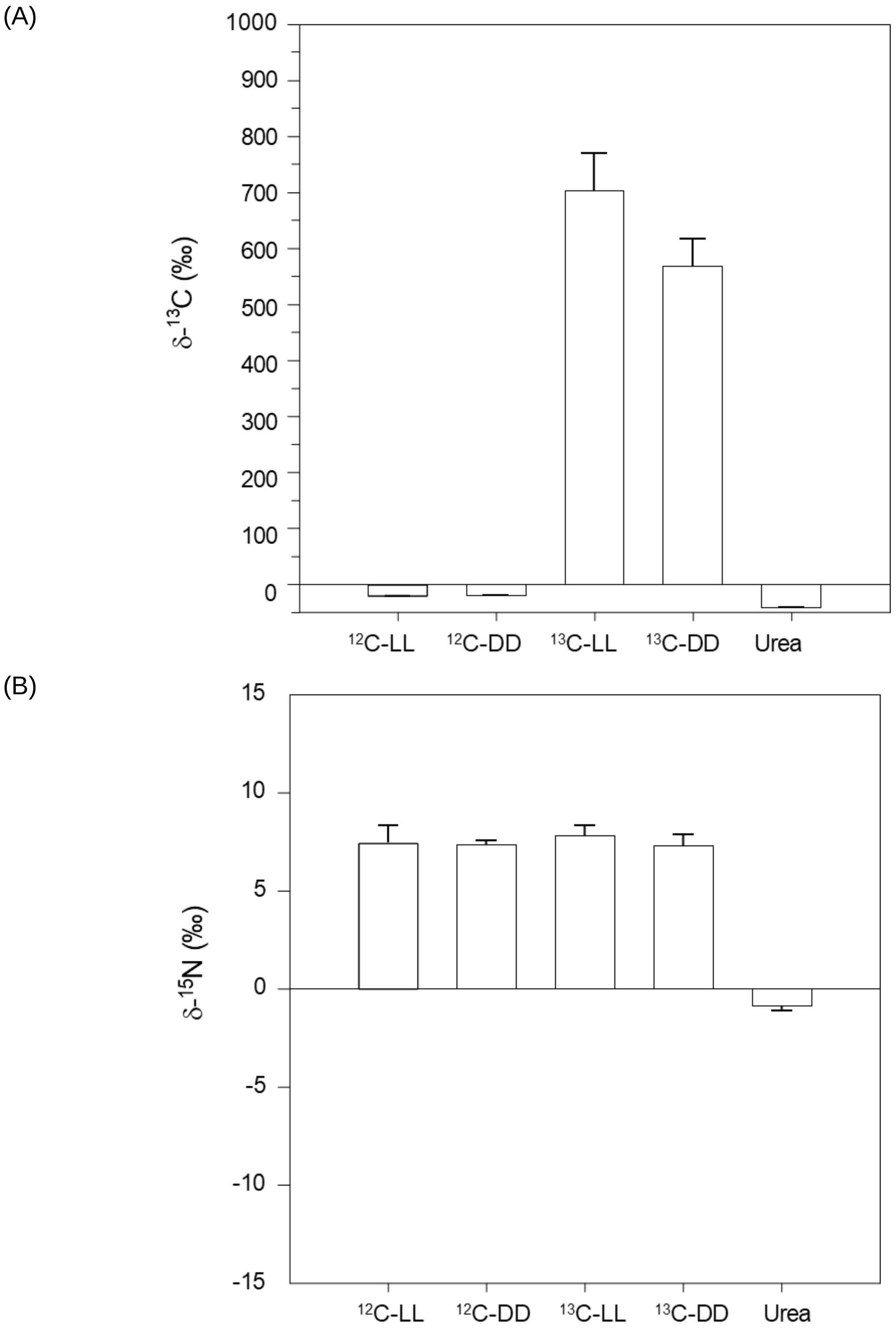

Fig. S2. Bar graphs for δ^{13}-C or δ^{15}-N of LL and DD cultures. Isotope-ratio mass sp ectrometry shown for harvested LL and DD cultures at late log phase cultures, and u rea were used as control. δ^{13}-C and δ^{13}-N were measured with 2.5 g of ^{13}C-$NaHCO_3$ added for each culture (300 ml).

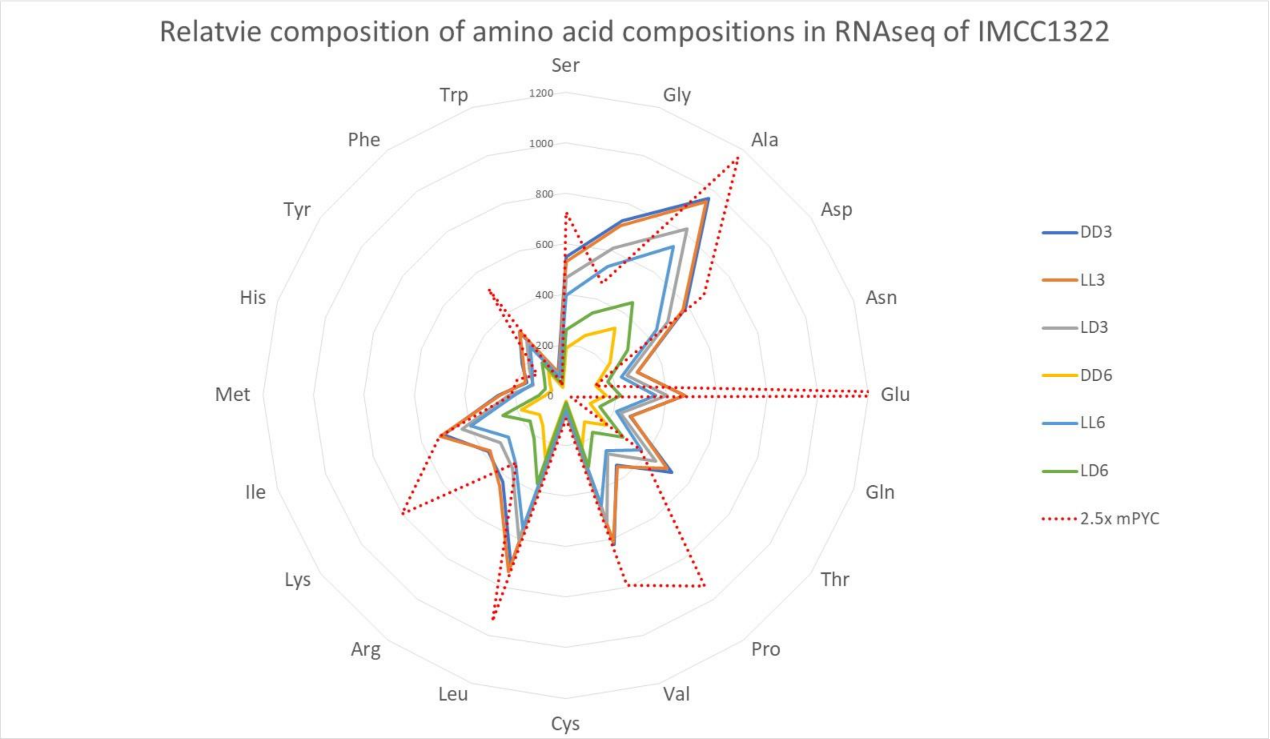

Fig. S3. Relative amino acid distribution in protein translation of IMCC1322. Proposed amino acids were from transcriptome data [1], and we assumed mRNA paralleled protein polymerization [23].

Fig. S4. KEGG diagram generated to show biosynthetic capability of amino acids on the genome of the strain IMCC1322. Green lines indicated metabolic paths that could be confirmed by genes for enzymes in IMCC1322. Numbers means micromolar concentration of free amino acids in 1x mPYC(Table S2 and Fig. S5). Red dashes mean that the provisional autotrophy of amino acids that include histidine, tryptophan, tyrosine, phenylalanine, and proline (see Table S3).

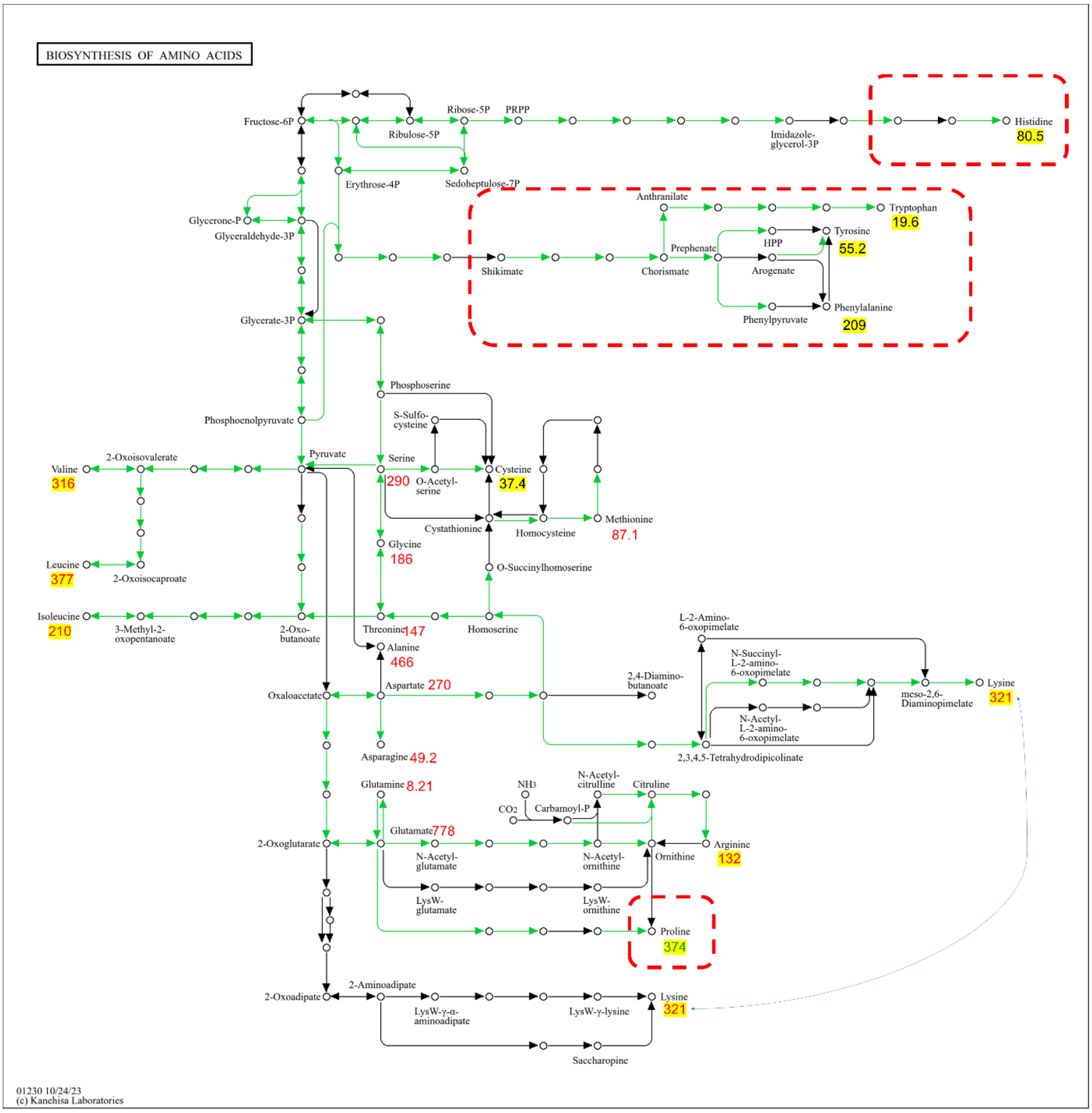

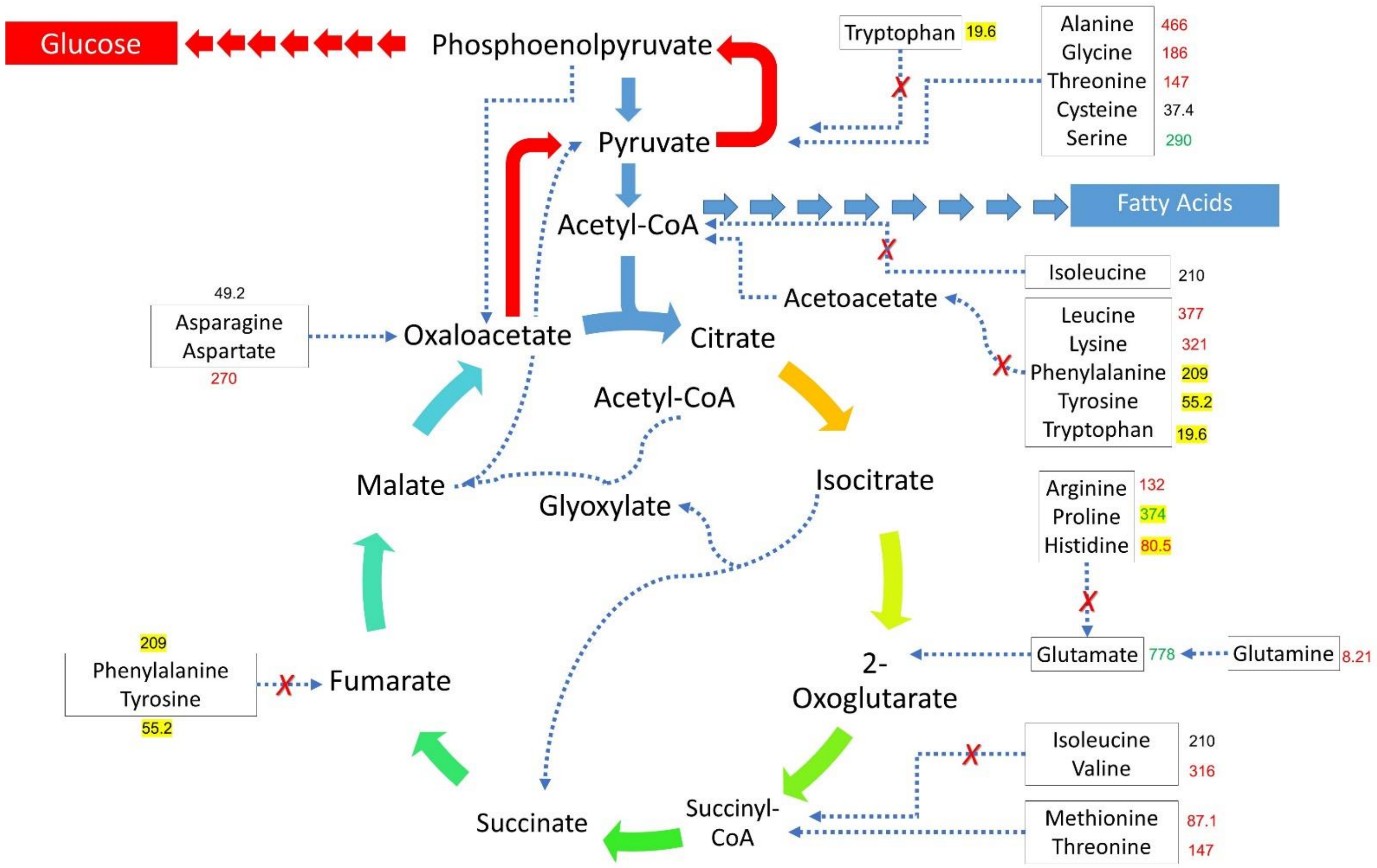

Fig. S5. Gluconeogenesis, glyoxylate bypass and TCA cycle for pyruvate formation. Amino acids(+100 μM) that tested positive in the growth test (Table S2) are indicated by red numbers(respective free [amino acid] in mPYC; μM). Amino acids(+100 μM) that tested weakly positive in the growth test are indicated by green numbers(respective free [amino acid] in mPYC; μM). Amino acids IMCC1322 cannot synthesize (Table S3) are indicated by yellow-background numbers(respective free [amino acid] in mPYC; μM).

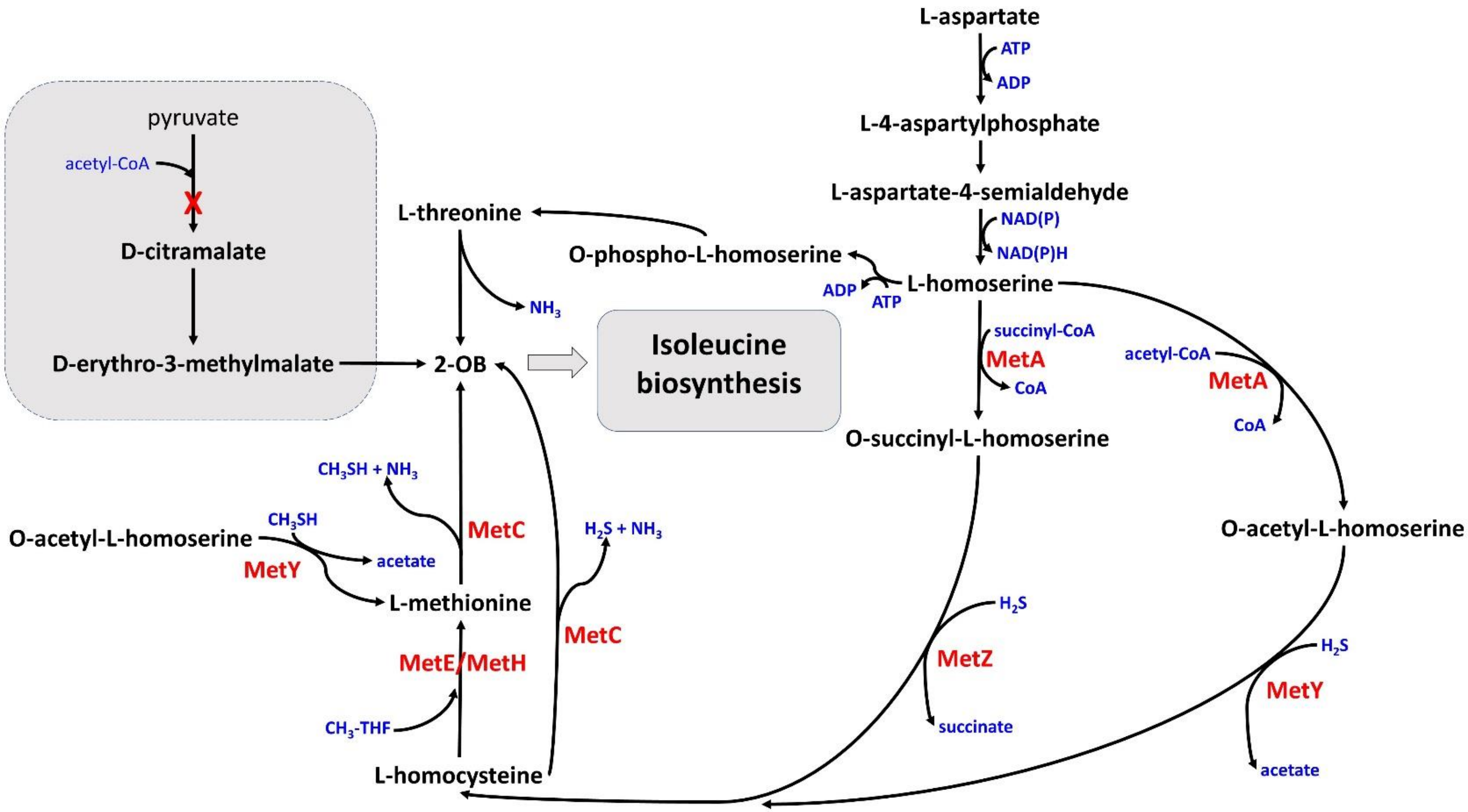

Fig. S6. Underground metabolism of isoleucine biosynthesis in strain IMCC1322. Isoleucine biosynthesis could start with pyruvate and 2-oxobutanoate(2OB), and 2OB could be simply formed from threonine and methionine in mPYC medium. Nonetheless, there would be intracellular L-homoserine and homocysteine pools for 2OB. Citramalate pathway for 2OB was impaired for one enzyme in IMCC1322. Template for the vignette were modified from the underground isoleucine biosynthesis in *E. coli* [9].

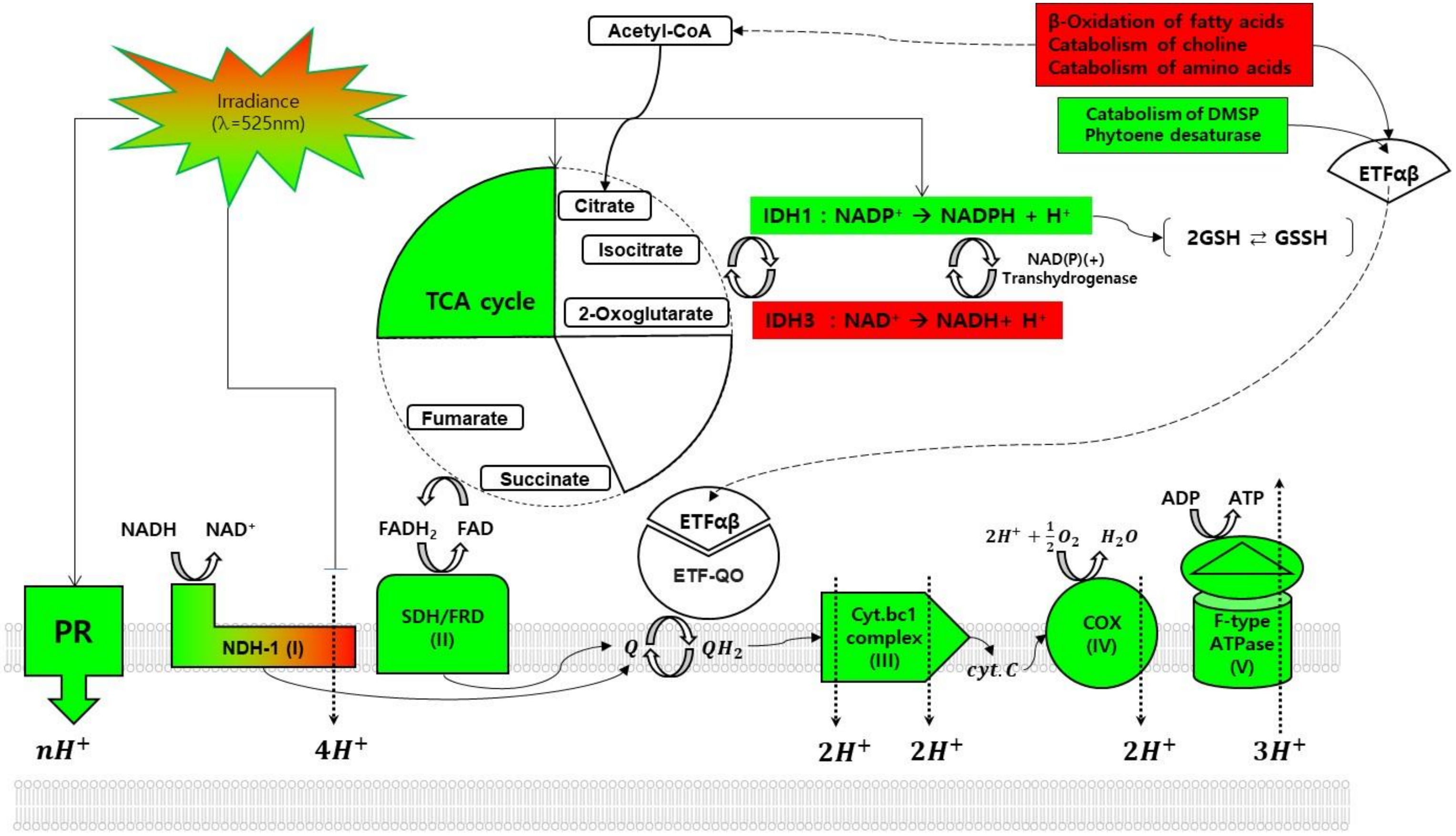

Fig. S7. A Vignette showing oxidative phosphorylation of stationary/death phase cultures of strain IMCC1322. Under light conditions transcription of anaplerotic TCA enzymes and NADPH by IDH1 were favored (green) except for Complex I. Dark condition upregulated Complex I and IDH3 for NADH (red) according to the previous report [1]. Linking of anabolism(NADPH for biomass production) and catabolism (NADH for ATP) may occur through membrane-bound NAD(P)(+)-transhydrogenase that depends on proton-motive force in periplasm [15].

Table S1. Isotope-ratio mass spectrometry of δ^{13}-C or δ^{15}-N of LL and DD cultures. No significant changes stable isotope signatures between LL and DD cultures(n=3; Student's t-test p<0.01).

Sample	δ^{13}-C (‰)	δ^{15}-N (‰)
Urea (control)	-40.84 ± 0.1693	-0.8967 ± 0.2446
LL culture	-19.99 ± 0.02383	6.942 ± 1.101
DD culture	-20.25 ± 0.09566	7.281 ± 0.2552
LL culture (+^{13}C-$NaHCO_3$)	690.2 ± 33.09	7.447 ± 0.4426
DD culture (+^{13}C-$NaHCO_3$)	609.9 ± 10.33	6.932 ± 0.6376

Table S2. Amino acids and ions in mPYC. Basal medium was prepared using 0.5 g/L each of Bacto™ Casamino Acids, Bacto™ Proteose Peptone No. 3, and Bacto™ Yeast Extract from BD. So-called mPYC is based on R2A [24, 25] whose formula w as modified by checking and omitting starch(~0.5 g/L), glucose(~2.76 mM), and then finally pyruvate(~ 2.65 mM). Turbidity raises in cultures were recorded as positive (+), weakly positive(w), and negative(−) growths when an amino acid was added ac cording to previous study [25]. Amino acids and other ions in the aged sea water ba sed 1x mPYC were calculated according to manufacturer's manual [26]. N.D. no dat a available.

Amino acid	μM(Free)	μM(Total)	Growth test (100 μM in mPYC)
Alanine	466	774	+*
Arginine	132	270	+
Asparagine	49.2	49.2	N.D.
Aspartate	270	579	+*
Cystine	18.7	18.7	−
Glutamate	778	1,132	w
Glutamine	8.21	8.21	+*
Glycine	186	726	+
Histidine	80.5	145	+
Isoleucine	210	389	N.D.
Leucine	377	560	+
Lysine	321	503	+*
Methionine	87.1	117	+*
Phenylalanine	209	294	N.D.
Proline	374	599	w*
Serine	290	247	w*
Threonine	147	202	+*
Tryptophan	19.6	19.6	N.D.
Tyrosine	55.2	88.3	N.D.
Valine	316	538	+†
**Sum**	**4.40×10^3**	**7.26×10^3**	
**Nitrogen**	**mg/L**	**mM**	
Total Nitrogen(TN)	0.176	12.5	
Amino Nitrogen(AN)	0.0955	6.82	
**AN/TN(%)**	**54.4%**	**54.6%**	
**Other inorganic ions**	**mg/L**	**μM**	
Phosphate	36.7	386	
Sulfate	5.05	52.6	
Cl^-	48.3	1.36	
Na^+	70.6	3,070	
K^+	24.6	629	
Mg^{++}	0.410	16.8	
Ca^{++}	0.161	4.00	

* : DL-amino acids were tested.

† : Positive in API-ZYM

Table S3. Energetic cost of canonical amino acids in mPYC. Energetic cost was adopted from data estimated using proteomes of *E. coli* and *Bacillus subtilis* [27] and heat of combustion of amino acids were from the publication by G. Livesey [28].

Amino acid	ATP consumption	NAD(P)H/FADH consumption	Total energetic cost of biosynthesis in ATP	Heat of combustion of amino acids (MJ/mole)	*de novo* synthesis of amino acid in IMCC1322	Degradation of amino acid in IMCC1322	Precursor metabolites
Glycine	2.3	4.7	11.7	0.97	+	+	3-phosphoglycerate
Serine	2.3	4.7	11.7	1.45	+	+	3-phosphoglycerate
Cysteine	7.3	8.7	24.7	2.23	+	+	3-phosphoglycerate
Glutamine	2.7	6.3	15.3	2.57	+	+	2-oxoglurate
Glutamate	3.7	6.3	16.3	2.25	+	+	2-oxoglurate
Proline	3.7	8.3	20.3	2.73	–	–	2-oxoglurate
Arginine	10.7	8.3	27.3	3.74	+	–	2-oxoglurate
Aspartate	1.3	5.7	12.7	1.61	+	+	oxaloacetate
Asparagine	3.3	5.7	14.7	1.93	+	+	oxaloacetate
Threonine	3.3	7.7	18.7	2.05	+	+	oxaloacetate
Methionine	9.7	12.3	34.3	2.78	+	+	oxaloacetate + cysteine - pyruvate
Lysine	4.3	13	30.3	3.68	+	–	oxaloacetate + pyruvate
Alanine	1	5.3	11.7	1.62	+	+	pyruvate
Valine	2	10.7	23.3	2.92	+	–	2 pyruvates
Leucine	2.7	12.3	27.3	3.58	+	–	2 pyruvates + acetyl-CoA
Isoleucine	4.3	14	32.3	3.58	+	–	pyruvate + oxaloacetate
Histidine	20.3	9	38.3	3.37	–	–	pentose phosphate
Tyrosine	13.3	18.3	50	4.44	–	–	2 phosphoenolpyruvates + erythrose phosphate
Phenylalanine	13.3	19.3	52	4.65	–	–	2 phosphoenolpyruvates + erythrose phosphate
Tryptophan	27.7	23.3	74.3	5.63	–	–	2 phosphoenolpyruvates + erythrose phosphate + phosphoribosyl pyrophosphate - pyruvate

References

1. Lee JH, Oh HM. 2024. Effects of Light and Dark Conditions on the Transcriptome of Aging Cultures of *Candidatus* Puniceispirillum marinum IMCC1322. *J Microbiol.* **62:** 297-314.
2. Verkhovsky MI, Bogachev AV. 2010. Sodium-translocating NADH:quinone oxidoreductase as a redox-driven ion pump. *Biochim Biophys Acta.* **1797:** 738-746.
3. Kimura H, Young CR, Martinez A, Delong EF. 2011. Light-induced transcriptional responses associated with proteorhodopsin-enhanced growth in a marine flavobacterium. *ISME J.* **5:** 1641-1651.
4. Blaza JN, Bridges HR, Aragao D, Dunn EA, Heikal A, Cook GM*, et al.* 2017. The mechanism of catalysis by type-II NADH:quinone oxidoreductases. *Sci Rep.* **7:** 40165.
5. Schuller JM, Birrell JA, Tanaka H, Konuma T, Wulfhorst H, Cox N*, et al.* 2019. Structural adaptations of photosynthetic complex I enable ferredoxin-dependent electron transfer. *Science.* **363:** 257-260.
6. Kravchuk V, Petrova O, Kampjut D, Wojciechowska-Bason A, Breese Z, Sazanov L. 2022. A universal coupling mechanism of respiratory complex I. *Nature.* **609:** 808-814.
7. Baradaran R, Berrisford JM, Minhas GS, Sazanov LA. 2013. Crystal structure of the entire respiratory complex I. *Nature.* **494:** 443-448.
8. Cavalcanti JH, Esteves-Ferreira AA, Quinhones CG, Pereira-Lima IA, Nunes-Nesi A, Fernie AR*, et al.* 2014. Evolution and functional implications of the tric

arboxylic acid cycle as revealed by phylogenetic analysis. *Genome Biol Evol.* **6:** 2830-2848.

9. Cotton CA, Bernhardsgrutter I, He H, Burgener S, Schulz L, Paczia N*, et al.* 2020. Underground isoleucine biosynthesis pathways in *E. coli*. *Elife.* **9:** e54207.

10. Mullins EA, Francois JA, Kappock TJ. 2008. A specialized citric acid cycle requiring succinyl-coenzyme A (CoA):acetate CoA-transferase (AarC) confers acetic acid resistance on the acidophile Acetobacter aceti. *J Bacteriol.* **190:** 4933-4940.

11. Kwong WK, Zheng H, Moran NA. 2017. Convergent evolution of a modified, acetate-driven TCA cycle in bacteria. *Nat Microbiol.* **2:** 17067.

12. Ensign SA. 2006. Revisiting the glyoxylate cycle: alternate pathways for microbial acetate assimilation. *Mol Microbiol.* **61:** 274-276.

13. Zheng J, Jia Z. 2010. Structure of the bifunctional isocitrate dehydrogenase kinase/phosphatase. *Nature.* **465:** 961-965.

14. **Nelson DL**, **Cox MM**. 2012. *Lehninger Principles of Biochemistry*, pp. 745-746. Ed. W. H. Freeman.

15. Sauer U, Canonaco F, Heri S, Perrenoud A, Fischer E. 2004. The soluble and membrane-bound transhydrogenases *UdhA* and *PntAB* have divergent functions in NADPH metabolism of *Escherichia coli*. *J Biol Chem.* **279:** 6613-6619.

16. Carini P, Steindler L, Beszteri S, Giovannoni SJ. 2013. Nutrient requirements for growth of the extreme oligotroph '*Candidatus* Pelagibacter ubique' HTCC1062 on a defined medium. *ISME J.* **7:** 592-602.

17. Tripp HJ. 2013. The unique metabolism of SAR11 aquatic bacteria. *J Microbio*

l. **51:** 147-153.

18. Palovaara J, Akram N, Baltar F, Bunse C, Forsberg J, Pedros-Alio C*, et al.* 2014. Stimulation of growth by proteorhodopsin phototrophy involves regulation of central metabolic pathways in marine planktonic bacteria. *Proc Natl Acad Sci U S A.* **111:** E3650-3658.
19. **Frerman FE**. 2013. Electron Transfer Flavoproteins, pp. 606-611. *In* Roberts GCK (ed.), *Encyclopedia of Biophysics*, Ed. Springer Berlin Heidelberg, Berlin, Heidelberg
20. Kim JJ, Miura R. 2004. Acyl-CoA dehydrogenases and acyl-CoA oxidases. Structural basis for mechanistic similarities and differences. *Eur J Biochem.* **271:** 483-493.
21. Engqvist MK, Esser C, Maier A, Lercher MJ, Maurino VG. 2014. Mitochondrial 2-hydroxyglutarate metabolism. *Mitochondrion.* **19 Pt B:** 275-281.
22. Buckel W, Thauer RK. 2013. Energy conservation via electron bifurcating ferredoxin reduction and proton/Na(+) translocating ferredoxin oxidation. *Biochim Biophys Acta.* **1827:** 94-113.
23. Russell JB, Cook GM. 1995. Energetics of bacterial growth: balance of anabolic and catabolic reactions. *Microbiol Rev.* **59:** 48-62.
24. Kang I, Oh HM, Kang D, Cho JC. 2013. Genome of a SAR116 bacteriophage shows the prevalence of this phage type in the oceans. *Proc Natl Acad Sci U S A.* **110:** 12343-12348.
25. Lee J, Kwon KK, Lim SI, Song J, Choi AR, Yang SH*, et al.* 2019. Isolation, cultivation, and genome analysis of proteorhodopsin-containing SAR116-clade strain *Candidatus* Puniceispirillum marinum IMCC1322. *J Microbiol.* **57:** 676-68

7.

26. Zimbro MJ, Power DA, Miller SM, Wilson GE, Johnson JA. 2018. Manual of Microbiological Culture Media, pp. 676-677. *Difco^{TM} & BBL^{TM} Manual*, 2ND Ed. Becton, Dickinson and Company, Sparks, Maryland 21152 USA

27. Akashi H, Gojobori T. 2002. Metabolic efficiency and amino acid composition in the proteomes of *Escherichia coli* and *Bacillus subtilis*. *Proc Natl Acad Sci U S A.* **99:** 3695-3700.

28. Livesey G. 1984. The energy equivalents of ATP and the energy values of food proteins and fats. *British Journal of Nutrition.* **51:** 15-28.
